# Supplementary material for: Meta-analysis of adverse events in clinical studies with antisense oligonucleotide therapies
Source: Mol Ther Nucleic Acids. 2026 Jun 8;37(3):102976. doi: 10.1016/j.omtn.2026.102976 (PMC13315839; doi:10.1016/j.omtn.2026.102976)
Supplement: Document S1. Figures S1 and S2 and Tables S1 and S4 [file mmc1.pdf]

OMTN, Volume *37*

Supplemental information

Meta-analysis of adverse events in clinical studies with antisense oligonucleotide therapies

Cisse Vermeer, Rindert R. Venema, Erwin Birnie, Marieke C. Bolling, Nine Knoers, Jeroen Bremer, and Peter C. van den Akker

PICO+S based search strategy as used for Pubmed. The inclusion of specific drugs in the search strategy did not guarantee the inclusion of relevant articles into the dataset.

("Oligonucleotides"[Mesh] OR "Oligonucleotide Probes"[Mesh] OR “oligonucleotide*”[tiab] OR “oligodeoxyribonucleotide*”[tiab] OR “oligoribonucleotide*”[tiab] OR “nusinersen”[tiab] OR “fomivirsen”[tiab] OR “pegaptanib”[tiab] OR “defibrotide”[tiab] OR “inotersen”[tiab] OR “eteplirsen”[tiab] OR “mipomersen”[tiab])
AND
("Drug-Related Side Effects and Adverse Reactions"[Mesh] OR "Long Term Adverse Effects"[Mesh] OR "adverse effects" [Subheading] OR “adverse*”[tiab] OR “safety”[tiab] OR “efficac*”[tiab] OR (“benefit”[tiab] *AND* “risk”[tiab]) OR "complications"[Subheading] OR "complication*"[tiab] OR "complicating"[tiab] OR "side effect*”[tiab] OR "contraindication*"[tiab] OR "contraindicated"[tiab] OR “toxicity”[tiab] OR "drug eruption*"[tiab] OR "toxic effect*"[tiab] OR "Toxic Actions"[Mesh] OR “toxic action*”[tiab] OR "poisoning"[tiab] OR "chemically induced"[Subheading] OR "chemically induced"[tiab] OR "Dose-Response Relationship, Drug"[Mesh] OR "Dose-Response Relationship“[tiab] OR “Treatment Failure”[tiab] OR "Treatment Failure"[Mesh])
AND
("Clinical Trial" [Publication Type] OR trial*[ti] OR “clinical study”[tiab] OR “clinical trial”[tiab])
NOT
("animals"[MeSH] NOT "humans"[MeSH])

Table S1, Significant differences in incidence rates between RNAse H and splice alteration adverse events with the respective 95% confidence intervals (95% CI). NEC: Not elsewhere classifiable.

ADVERSE EVENT	SPLICE STUDIES	SPLICE PREVALENCE	95% CI LOWER	95% CI UPPER	RNASE H STUDIES	RNASE H PREVALENCE	95% CI LOWER	95% CI UPPER
ASTHENIC CONDITIONS	3	0.10	0.04	0.23	38	0.30	0.23	0.38
HEADACHES NEC	15	0.27	0.22	0.33	31	0.17	0.14	0.21
UPPER RESPIRATORY TRACT INFECTIONS	17	0.32	0.21	0.45	21	0.01	0.00	0.06
COUGHING AND ASSOCIATED SYMPTOMS	14	0.29	0.20	0.40	11	0.09	0.07	0.13
COAGULATION AND BLEEDING ANALYSES	4	0.05	0.02	0.14	7	0.53	0.31	0.73
UPPER RESPIRATORY TRACT SIGNS AND SYMPTOMS	9	0.20	0.12	0.31	5	0.07	0.04	0.12
POTASSIUM IMBALANCE	3	0.41	0.25	0.59	3	0.12	0.07	0.20
RATE AND RHYTHM DISORDERS NEC	8	0.10	0.06	0.18	3	0.02	0.01	0.06
VIRAL INFECTIONS NEC	7	0.16	0.09	0.27	3	0.05	0.04	0.07
DERMATITIS AND ECZEMA	13	0.14	0.10	0.21	2	0.02	0.01	0.09

Table S2, Incidence of all reported events in three or more studies resulting from ASO treatment. with the respective 95% confidence intervals (95% CI). NEC: Not elsewhere classifiable

For Table S2 see supplemental spreadsheet *TableS2.xlsx*

***Table S3*, *Risk of bias analysis per article*.** *Bias assessment per category of bias is made with an assessment of low (green) some (orange) of high (red) risk of bias. Entries marked with * were separately assessed because of different study formats conducted within the same article*

For Table S3 see supplemental spreadsheet *TableS3.xlsx*

Table S4*, *Variables used in the meta regression that were performed for each analysis. *In each analysis a study was assigned a group for every variable.*

REGRESSION VARIABLE	GROUP 1	GROUP 2	GROUP 3	GROUP 4	GROUP 5
**CHEMISTRY**	2'-Ome-PS	2'-MOE-PS	DNA oligonucleotides	PMO	Other chemistries
**SUBJECT HEALTH**	Healthy	Cancer	Muscle diseases	Eye diseases	Other disease
**ADMINISTRATION ROUTE**	Intravenous	Intrathecal	Subcutaneous	Other administration routes	
**MODE OF ACTION**	RNAse H	Splice switching	Other mode of action		
**SUBJECT AGE**	0-18	19-64	65+		
**PUBLICATION DATE**	1990-2010	2011-2017	2018-2023		
**PLACEBO CONTROLLED**	Yes	No			

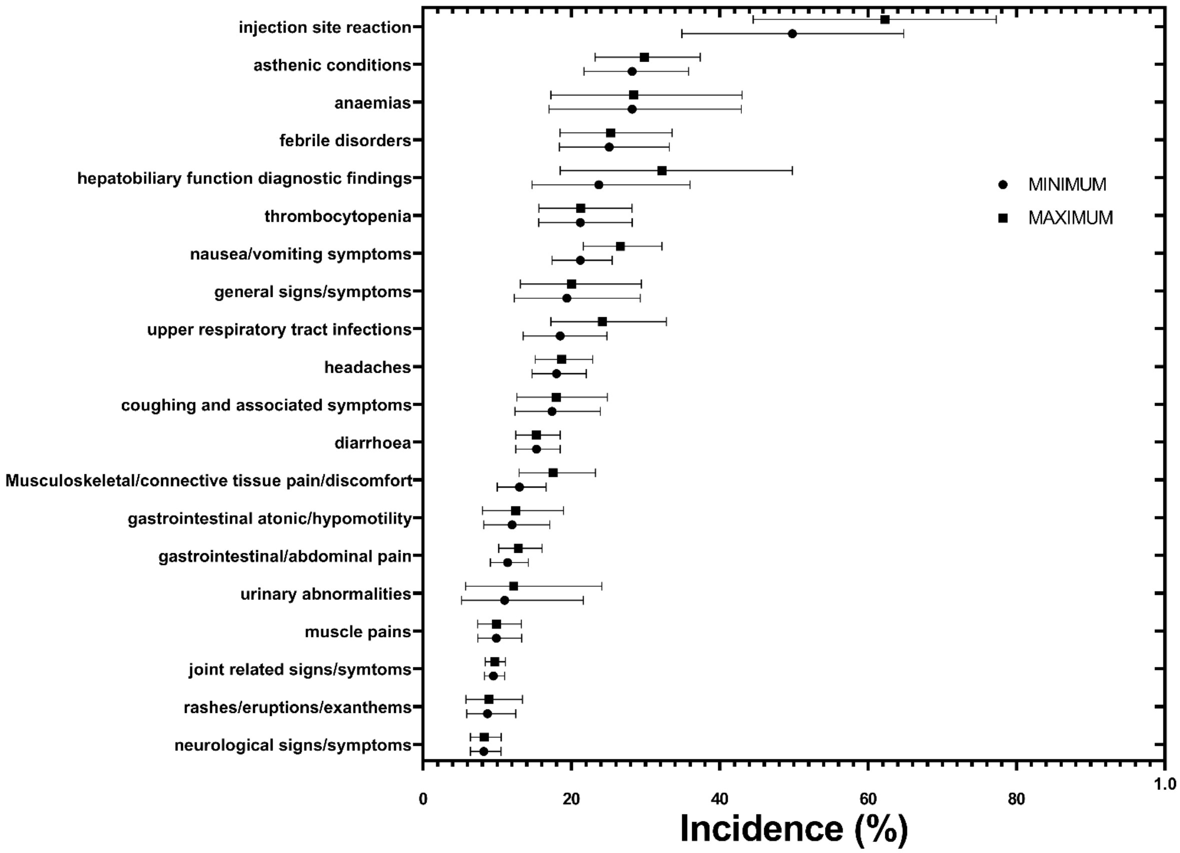

Figure S1, Differences in incidence rates of the top 20 most often reported adverse events*, comparing minimum and maximum event counts. There are no significant differences between minimum and maximum counts for any of the measured events.*

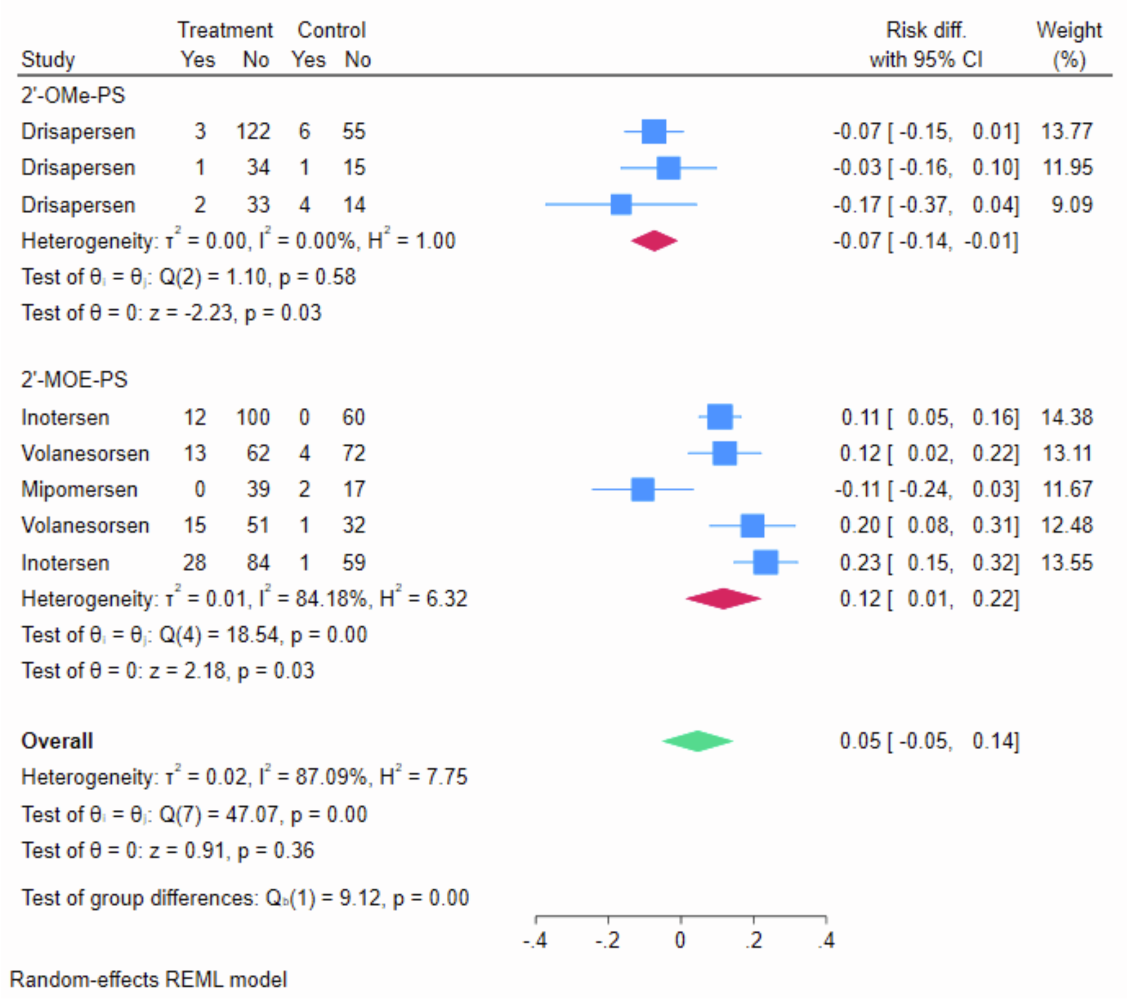

Figure S2, Forest plot of merged thrombocytopenia-related AEs. *These AEs include the terms "platelet disorders", "platelet disorders NEC (Not elsewhere clarified)", "thrombocytopenia", "platelet analyses" and "coagulation and bleeding analyses". Overall effect size is shown by the green diamond. Effect sizes per ASO chemistry subgroup are shown as red diamonds, demonstrating that coagulation-related events have a statistically significant higher risk difference in patient groups treated with 2'MOE PS ASOs compared to 2'OMe PS ASOs. The effect size per study is presented as a blue box with corresponding confidence intervals (95% CI). This forest plot shows that the overall effect size is not significant (θi: $p>0.05$) but the effect sizes for each subgroup are significant (θ: $p<0.05$). Measures of heterogeneity (T^2, I^2, H^2) point to show reduced heterogeneity in each of the subgroups.*
