# Supplementary material for: Meta-analysis of adverse events in clinical studies with antisense oligonucleotide therapies
Source: Mol Ther Nucleic Acids. 2026 Jun 8;37(3):102976. doi: 10.1016/j.omtn.2026.102976 (PMC13315839; doi:10.1016/j.omtn.2026.102976)
Supplement: Document S2. Article plus supplemental information [file mmc4.pdf]

Molecular Therapy
Nucleic Acids
Review

Meta-analysis of adverse events in clinical studies with antisense oligonucleotide therapies

Cisse Vermeer,[1] Rindert R. Venema,[2] Erwin Birnie,[1] Marieke C. Bolling,[2] Nine Knoers,[1] Jeroen Bremer,[2] and Peter C. van den Akker[1]

[1]University of Groningen, University Medical Center Groningen, Department of Genetics, 9700RB Groningen, the Netherlands; [2]University of Groningen, University Medical Center Groningen, Department of Dermatology, 9700RB Groningen, the Netherlands

Antisense oligonucleotides (ASOs) are increasingly being used as a platform to target various diseases. Currently, there are 13 USFDA- or EMA-approved ASO therapies. Adverse events resulting from ASO treatments are assessed on a per drug basis, but for many ongoing ASO developments targeting N-of-1 mutations, conventional randomized clinical trials to assess safety cannot be performed. Here, we conducted a systematic review and meta-analysis of adverse events in clinical studies conducting trials of ASO therapies. The study aims to provide better insight into the safety aspects of ASO design choices and to summarize knowledge of the safety aspects of ASOs so that we can better inform researchers and physicians of possible adverse events that can occur during (N-of-1) ASO treatments. Our results provide a list of common adverse events and event rates obtained from pooled data from both approved and non-approved ASO drugs, and we include recommendations that can provide insights into the nature of ASO-induced adverse events in future trials.

INTRODUCTION

Antisense oligonucleotide (ASO) therapies are a promising personalized medicine approach for (rare) genetic diseases,[1] but these therapies are also increasingly being explored and applied to treat common diseases.[2] ASOs belong to the category of RNA therapeutics and are short (usually ∼20 bases) strands of synthetic nucleotides. ASOs are designed to remedy a disease by targeting an mRNA of interest. These RNAs can, for example, be targeted through the RNase-H or splice modulating mechanisms. In the RNase-H mechanism, the ASO can modulate gene expression and silence the RNA through degradation. The target mRNA sequence is hybridized by the ASO, directing RNase-H1-mediated cleavage to the target site, thereby reducing mRNA expression.[3] Alternatively, ASOs can correct splicing defects and/or restore gene function[1] through splice modification mechanics. Splicing ASOs can be designed to interfere with splicing, causing the exclusion or inclusion of exons, introns, or pseudoexons, which alter the expressed protein (or its expression levels).[4] The targeted approach of designing an ASO sequence toward a specific variant makes ASOs a suitable tool to develop and potentially treat N-of-1 variants, which has been successfully demonstrated in the past by the well-known example of milasen.[5]

The next step in ASO design is chemistry. Various chemical modifications to ASOs have been developed and tested in clinical trials. The earliest developments used DNA oligonucleotides, usually with a modification in the backbone of the oligo, such as the phosphorothioate (PS) linker. In more recent developments modifications in the nucleotide ribose such as 2’-*O*-methyl (2’-OMe), 2’-*O*-methoxyethyl (2’-MOE), and phosphorodiamidate morpholino oligomers (PMOs) are common.[6–8]

Finally, the route of administration and delivery are also of main importance to the safety and efficacy of ASOs. The administration route is often determined by the target tissue (e.g., intrathecal administration for targeting the central nervous system). The three most common administration routes of ASOs are intravenous infusion, subcutaneous injection, and intrathecal injection. Further development in ASO delivery is ongoing; a well-known example of a significant development in targeted ASO delivery is the GalNAc conjugation directing ASOs to the liver.[9]

Together, ASO chemistry, mode of action, and administration route (hereinafter referred to as “ASO design properties”) influence not only ASO efficacy but also its safety profile.[10] Development of a safe and effective ASO treatment, therefore, requires optimization of the sequence, chemistry, and delivery route[11] and a subsequent careful evaluation on the balance between efficacy and safety. Given all the variables inherent to ASO design, it is clear that ASOs are a highly heterogeneous group of molecules. This heterogeneity makes it challenging to assign a generalized safety profile to every ASO.

Nonetheless, despite their heterogeneity, ASOs are associated with some common adverse events (AEs), with AEs like liver damage or

https://doi.org/10.1016/j.omtn.2026.102976.

Correspondence: Jeroen Bremer, Department of Dermatology, University Medical Center Groningen, PO Box 30.001, 9700 RB Groningen, the Netherlands.
E-mail: j.bremer@umcg.nl

Published by Elsevier Inc. on behalf of The American Society of Gene and Cell Therapy.
This is an open access article under the CC BY license (http://creativecommons.org/licenses/by/4.0/).

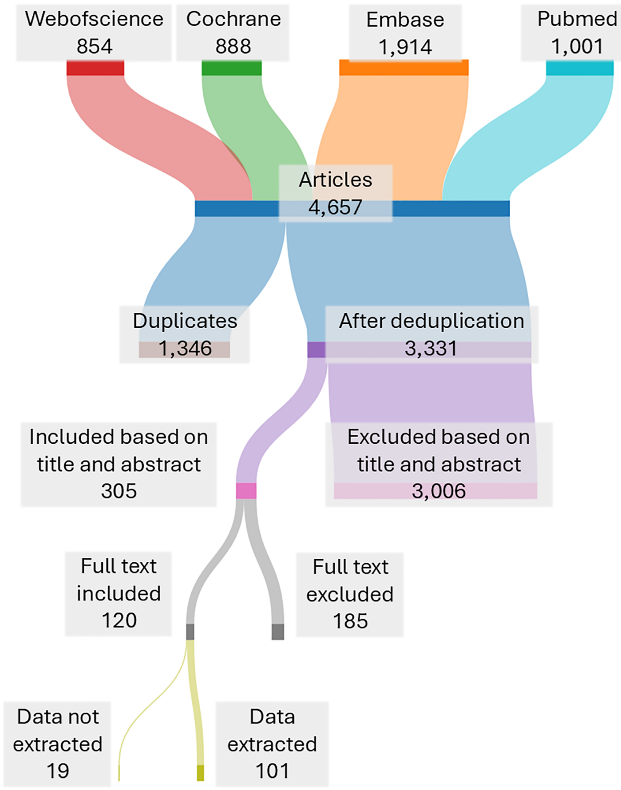

Figure 1. Flowchart detailing the literature selection process
The initial literature search was conducted in literature search engines Web of Science, Cochrane, Embase, and PubMed. After removal of duplicate articles, the remainder was screened based on title and abstract. Articles included subsequently were then assessed based of the full text, which resulted in 101 articles being included into the dataset.

thrombocytopenia repeatedly reported.[10] ASO toxicities can be divided into sequence-dependent and sequence-independent toxicities.[12] Sequence-dependent AEs are sequence driven and therefore potentially different for every ASO. Sequence-independent AEs are not sequence-specific and are commonly divided into four subcategories[13–15]: (1) accumulation in the kidney and liver tissues, (2) complement activation, (3) pro-inflammatory effects, and (4) thrombocytopenia (notably, some studies argue that thrombocytopenia can also be sequence-derived[16,17]).

For researchers developing ASOs, physicians treating patients, and patients, insight into AEs that could result from ASO therapies is crucial for individual treatment setting and benefit-risk assessment of ASO treatments. Safety information is available for drugs approved by the FDA or European commission on a product specific basis.[18] However, for small cohorts ($N = 1$ or few) targeting patients with (ultra)rare diseases, it is challenging to obtain sufficient safety data, because regular clinical trials cannot be performed. On top of this, a quantified risk profile for ASOs in general is lacking in current literature, in part because clinical studies assessing ASOs are highly heterogeneous.[13–15,19]

To better understand ASO-induced AEs, we conducted a systematic review and meta-analysis of AEs reported in published ASO-in-human studies. We included data on both USFDA- or European commission-approved drugs and on compounds tested in humans but not approved or submitted for review. This study aims to provide: (1) quantitative insight into AEs associated with specific ASOs and (2) safety information that can be used to inform researchers and physicians of possible AEs as part of (N-of-1) ASO treatments.

RESULTS

A literature search on adverse events in patients treated with antisense oligonucleotides results in a large dataset of 101 studies

Our meta-analysis is the first to report on pooled AE rates in ASO-treated as well as placebo-controlled studies. The major strength of this meta-analysis lies in the between-study analyses of AE data from 101 clinical studies of both approved and non-approved/submitted drugs.

Our search strategy was developed according to the PICO+S inclusion criteria principle for literature searches.[20] Our inclusion criteria based on PICO+S were "human," "antisense oligonucleotide," "adverse event," and "clinical study". The criteria encompassed a human patient population, with no further exclusion criteria. Treatment criteria included treatment with ASOs of any chemistry, with no restrictions on treatment period. Both placebo-controlled and uncontrolled studies were included. Only quantitative AE data was included. PubMed MeSH terms were used to construct a search strategy encompassing all inclusion criteria to retrieve as much relevant literature as possible (see supplemental information). Eight manually retrieved articles were used as controls to validate our search strategy. This search strategy was then adapted to the Embase, Cochrane, and Web of Science search engines, yielding 4,657 articles (Figure 1). After deduplication, 3,311 articles remained. Of these articles, 305 met the inclusion criteria. The full text of these articles was then screened to check whether they reported AEs in a quantitative format (e.g., tables in the article or supplements or a clinicaltrials.gov entry), resulting in 120 eligible articles. For 19 of these 120 articles, other conditions were present that did not lead to the inclusion of these articles, such as reporting methods that that did not provide quantifiable data or reporting combination treatment trials. This left 101 articles meeting all the criteria for data retrieval (Table 1), with 47 describing placebo-controlled studies. The data extracted from the 101 articles included a total of 6,163 patients, of which 4,901 were treated with an ASO and 1,262 with placebo (either saline injection/infusion or sham). AE rates were extracted and categorized according to the Medical Dictionary for Regulatory Activities (MedDRA)[122] hierarchy of terms, with the top tier consisting of 28 system organ classes (SOCs) (e.g., lung, skin, and heart). SOCs were then subdivided into 378 higher-level terms (HLTs) specifying the type of AE. From this overall dataset, subgroups could be made based on the ASO

Table 1. Study information of all articles used for data extraction

Study	Administered ASO	Injection route	Mode of action	Chemistry	Placebo-controlled	Treated	Placebo	Population type
1[21]	IONIS-TTRrx	subcutaneous	RNase H	2'-MOE-PS	yes	39	10	healthy
2[22]	nusinersen	intrathecal	splice alteration	2'-MOE-PS	yes	20	7	spinal muscular atrophy
3[23]	aprinocarsen	intravenous	RNase H	DNA-PS	no	15	–	cancer
4[24]	aprinocarsen	intravenous	RNase H	DNA-PS	no	36	–	cancer
5[25]	mipomersen	subcutaneous	RNase H	2'-MOE-PS	yes	80	10	hyperlipidemia
6[26]	OL(1)P53	intravenous	RNase H	DNA-PS	no	5	–	cancer
7[27]	radavirsen	intravenous	splice alteration	PMO	yes	42	14	healthy
8[28]	inotersen	subcutaneous	RNase H	2'-MOE-PS	yes	112	60	hereditary transthyretin amyloidosis
9[29]	EZN-4176	intravenous	RNase H	LNA	no	22	–	cancer
10[30]	GEM231	intravenous	RNase H	2'-OMe-PS	no	14	–	cancer
11[31]	apatorsen	intravenous	RNase H	2'-MOE-PS	no	42	–	cancer
12[32]	nusinersen	intrathecal	splice alteration	2'-MOE-PS	no	28	–	spinal muscular atrophy
13[33]	sepofarsen	intravitreal	splice alteration	2'-OMe-PS	no	11	–	leber congenital amaurosis
14[34]	eteplirsen	intravenous	splice alteration	PMO	no	19	–	Duchenne muscular dystrophy
15[35]	viltolarsen	intravenous	splice alteration	PMO	no	16	–	Duchenne muscular dystrophy
16[36]	viltolarsen	intravenous	splice alteration	PMO	yes	27	5	Duchenne muscular dystrophy
17[37]	ISIS 5132	intravenous	RNase H	DNA-PS	no	22	–	cancer
18[38]	ISIS 3512/ISIS 5132	intravenous	RNase H	DNA-PS	no	37	–	cancer
19[39]	ISIS 5132	intravenous	RNase H	DNA-PS	no	34	–	cancer
20[40]	MG98	intravenous	RNase H	2'-OMe-PS	no	14	–	cancer
21[41]	nusinersen	intrathecal	splice alteration	2'-MOE-PS	no	25	–	spinal muscular atrophy
22[42]	GTI-2040	intravenous	RNase H	DNA-PS	no	26	–	cancer
23[43]	eluforsen	inhalation	unknown	2'-OMe-PS	yes	61	9	cystic fibrosis
24[44]	donidalorsen$^{\Delta}$	intrathecal	splice alteration	2'-MOE-PS	yes	21	6	angioedema
25[45]	nusinersen	intrathecal	splice alteration	2'-MOE-PS	no	16	–	spinal muscular atrophy
26[46]	nusinersen	intrathecal	splice alteration	2'-MOE-PS	yes	80	41	spinal muscular atrophy
27[47]	mipomersen	subcutaneous	RNase H	2'-MOE-PS	yes	63	21	healthy
28[48]	drisapersen	subcutaneous	splice alteration	2'-OMe-PS	yes	15	5	Duchenne muscular dystrophy
29[49]	RO7062931$^{\Delta}$	subcutaneous	RNase H	LNA	yes	44	15	chronic hepatitis B
30[50]	volanesorsen	subcutaneous	RNase H	2'-MOE-PS	yes	41	16	hypertrigliyceridemia
31[51]	GEM231	intravenous	RNase H	2'-OMe-PS	no	14	–	cancer

(*Continued on next page*)

Table 1. *Continued*

Study	Administered ASO	Injection route	Mode of action	Chemistry	Placebo-controlled	Treated	Placebo	Population type
32[52]	drisapersen	subcutaneous	splice alteration	2’-OMe-PS	yes	125	61	Duchenne muscular dystrophy
33[53]	drisapersen	subcutaneous	splice alteration	2’-OMe-PS	no	12	–	Duchenne muscular dystrophy
34[54]	drisapersen	subcutaneous	splice alteration	2’-OMe-PS	no	12	–	Duchenne muscular dystrophy
35[55]	volanesorsen	subcutaneous	RNase H	2’-MOE-PS	yes	75	38	multifactorial chylomicronaemia syndrome
36[56]	nusinersen	intrathecal	splice alteration	2’-MOE-PS	no	173	–	spinal muscular atrophy
37[57]	GSK3389404[Δ]	subcutaneous	RNase H	2’-MOE-PS	yes	42	14	healthy
38[58]	LY2275796	intravenous	RNase H	2’-MOE-PS	no	30	–	cancer
39[59]	trabedersen	convection enhanced delivery	RNase H	DNA-PS	yes	90	45	cancer
40[60]	EZN-2968	intravenous	RNase H	LNA	no	10	–	cancer
41[61]	GS-101	eye drop	RNase H	DNA-PS	no	14	–	healthy
42[62]	mipomersen	subcutaneous	RNase H	2’-MOE-PS	yes	29	7	dyslipidemia
43[63]	MG98	intravenous	RNase H	2’-OMe-PS	no	23	–	cancer
44[64]	viltolarsen	intravenous	splice alteration	PMO	no	10	–	Duchenne muscular dystrophy
45[65]	viltolarsen	intravenous	splice alteration	PMO	no	16	–	Duchenne muscular dystrophy
46[66]	VEGF-AS	intravenous	RNase H	DNA-PS	no	50	–	cancer
47[67]	IONIS-DGAT2rx	subcutaneous	RNase H	2’-MOE-PS	yes	29	26	non-alcoholic fatty liver disease
48[68]	ISIS 2302	intravenous	RNase H	DNA-PS	no	32	–	rheumatoid arthritis
49[69]	ISIS 3152	intravenous	RNase H	DNA-PS	no	16	–	cancer
50[70]	eteplirsen	intravenous	splice alteration	PMO	no	79	–	Duchenne muscular dystrophy
51[71]	drisapersen	subcutaneous	splice alteration	2’-OMe-PS	yes	35	16	Duchenne muscular dystrophy
Study	Administered ASO	Injection route	Mode of action	Chemistry	Placebo-controlled	Treated	Placebo	Patient health
52[72]	mipomersen	subcutaneous	RNase H	2’-OMe-PS	yes	39	19	familial hypercholesterolemia
53[73]	eteplirsen	intravenous	splice alteration	PMO	yes	20	4	Duchenne muscular dystrophy
54[74]	eteplirsen	intravenous	splice alteration	PMO	yes	8	4	Duchenne muscular dystrophy
55[75]	nusinersen	intrathecal	splice alteration	2’-MOE-PS	yes	84	42	spinal muscular atrophy
56[76]	tofersen	intrathecal	RNase H	2’-MOE-PS	yes	38	12	amyotrophic lateral sclerosis
57[77]	tofersen	intrathecal	RNase H	2’-MOE-PS	yes	24	8	amyotrophic lateral sclerosis
58[78]	mongersen	oral	RNase H	DNA-PS	no	15	–	Crohn’s disease
59[79]	IONIS-AGT-LRx	subcutaneous	RNase H	2’-MOE-PS	yes	70	16	healthy
60[80]	G3139	intravenous	RNase H	DNA-PS	no	35	–	cancer
61[81]	ISIS 3521	intravenous	RNase H	DNA-PS	no	36	–	cancer
62[82]	ISIS-CRPrx	intravenous	RNase H	2’-MOE-PS	yes	23	12	healthy
63[83]	G3139	intravenous	RNase H	DNA-PS	no	40	–	cancer

(*Continued on next page*)

Table 1. *Continued*

Study	Administered ASO	Injection route	Mode of action	Chemistry	Placebo-controlled	Treated	Placebo	Patient health
64[84]	ISIS 5132	intravenous	RNase H	DNA-PS	no	19	–	cancer
65[85]	ISTH0036	intravitreal	RNase H	DNA-PS	no	12	–	primary open angle glaucoma
66[86]	MG98	intravenous	RNase H	2’-*O*Me-PS	no	33	–	cancer
67[87]	mipomersen	subcutaneous	RNase H	2’-MOE-PS	yes	3	4	familial hypercholesterolemia
68[88]	mipomersen	subcutaneous	RNase H	2’-MOE-PS	yes	34	17	familial hypercholesterolemia
69[89]	AEG35156	intravenous	RNase H	2’-*O*Me-PS	no	38	–	cancer
70[90]	ISIS 3521	intravenous	RNase H	DNA-PS	no	26	–	cancer
71[91]	mipomersen	subcutaneous	RNase H	2’-MOE-PS	yes	207	103	familial hypercholesterolemia
72[92]	AZD9150	intravenous	RNase H	unknown	no	30	–	cancer
73[93]	LR-3280	intracoronary	RNase H	DNA-PS	yes	52	26	coronary restenosis
74[94]	ISIS 5132	intravenous	RNase H	DNA-PS	no	22	–	cancer
75[95]	ISIS 5132	intravenous	RNase H	DNA-PS	no	22	–	cancer
76[96]	sepofarsen	intraocular	splice alteration	2’-*O*Me-PS	no	22	–	Leber congenital amaurosis
77[97]	mongersen	oral	RNase H	DNA-PS	yes	527	174	Crohn’s disease
78[98]	mipomersen	subcutaneous	RNase H	2’-MOE-PS	no	141	–	familial hypercholesterolemia
79[99]	eluforsen	intranasal	unknown	2’-*O*Me-PS	no	18	–	cystic fibrosis
80[100]	golodirsen	intravenous	splice alteration	PMO	no	25	–	Duchenne muscular dystrophy
81[101]	mipomersen	subcutaneous	RNase H	2’-MOE-PS	yes	83	41	familial hypercholesterolemia
82[102]	ISIS 5132	intravenous	RNase H	DNA-PS	no	29	–	cancer
83[103]	MG98	intravenous	RNase H	2’-*O*Me-PS	no	19	–	cancer
84[104]	LY2181308	intravenous	RNase H	DNA-PS	no	14	–	cancer
85[105]	olezarsen[Δ]	subcutaneous	RNase H	2’-MOE-PS	yes	90	24	atherosclerotic cardiovascular disease
86[106]	CDR132L	intravenous	RNase H	LNA-PS	yes	20	8	heart failure
87[107]	mipomersen	subcutaneous	RNase H	2’-MOE-PS	yes	105	52	familial hypercholesterolemia
88[108]	ATL1103	subcutaneous	RNase H	2’-MOE-PS	no	26	–	acromegaly
89[109]	ISIS 388626	subcutaneous	RNase H	2’-MOE-PS	yes	40	13	healthy
90[110]	AKCEA-TTR-LRx[Δ]	intravenous	RNase H	2’-MOE-PS	yes	39	6	healthy
91[111]	mipomersen	subcutaneous	RNase H	2’-MOE-PS	yes	10	11	familial hypercholesterolemia
92[112]	mipomersen	subcutaneous	RNase H	2’-MOE-PS	yes	21	12	familial hypercholesterolemia
93[113]	drisapersen	subcutaneous	splice alteration	2’-*O*Me-PS	yes	35	18	Duchenne muscular dystrophy
94[114]	casimersen	subcutaneous	splice alteration	PMO	yes	12	4	Duchenne muscular dystrophy
95[115]	ISIS-CRPRx	subcutaneous	RNase H	2’-MOE-PS	yes	39	12	rheumatoid arthritis
96[116]	G3139	subcutaneous	RNase H	DNA-PS	no	22	–	cancer
97[117]	MG98	intravenous	RNase H	2’-*O*Me-PS	no	17	–	cancer
98[118]	volanesorsen	subcutaneous	RNase H	2’-MOE-PS	yes	33	33	familial chylomicronemia
99[119]	alicaforsen	intravenous	RNase H	DNA-PS	no	331	–	Crohn’s disease
100[120]	alicaforsen	intravenous	RNase H	DNA-PS	yes	198	101	Crohn’s disease
101[121]	inotersen	subcutaneous	RNase H	2’-MOE-PS	yes	112	60	TTR amyloid polyneuropathy

Δ, this drug is conjugated with GalNAc; LNA, locked nucleic acid chemistry; DNA-PS, DNA oligo with phosphorothioate linker; 2’-*O*Me-PS, 2’-*O*-methyl chemistry with phosphorothioate linker; 2’-MOE-PS, 2’-*O*-methoxyethyl chemistry with phosphorothioate linker; PMO, phosphorodiamidate morpholino oligomers.

chemistry, administration route or mode of action, as well as whether as study was placebo-controlled or not. Table 2 summarizes this extracted data. However, performing analysis on the heterogeneous dataset required addressing any potential bias or confounding variables that result from this dataset.

Comparison between minimum and maximum adverse event counts validates the dataset

Because of differences in reporting and terminology used between studies, patients may be counted multiple times within one report. For example, one report of nausea and one report of vomiting

Table 2. Summary of the studies, patients, and ASO properties included in this meta-analysis

Number of studies		101	Placebo-controlled studies				47
Total patients		6,163	patients in placebo studies				4,298
Number of drugs included		49	number of drugs in placebo studies				25
ASO-treated patients		4,901	ASO-treated in placebo studies				3,036
Estimated adverse events (minimum)		12,622	placebo-treated patients				1,262
Estimated adverse events (maximum)		14,566	–				–
Difference between minimum and maximum		1,944	–				–
	Studies	Treated		Studies	Treated	Placebo	Placebo:treatment ratio (%)
Chemistry							
2'-MOE-PS studies	39	2,206	placebo-controlled MOE studies	31	1,725	753	43.6
2'-OMe-PS studies	18	518	placebo-controlled OMe studies	5	271	109	40.2
DNA-PS studies	28	1,777	placebo-controlled DNA studies	4	867	346	39.9
PMO studies	11	274	placebo-controlled PMO studies	5	109	31	28.4
Administration route							
Intravenous studies	48	1,743	placebo-controlled intravenous studies	9	425	170	40.0
Intrathecal studies	10	509	placebo-controlled intrathecal studies	6	267	116	43.4
Subcutaneous studies	34	1,875	placebo-controlled subcutaneous studies	29	1,493	672	45.0
Mode of action							
RNase H studies	73	1,918	placebo-controlled RNase H studies	33	2,451	1,026	41.9
Splice alteration studies	27	988	placebo-controlled splice alteration studies	13	524	227	43.3

DNA-PS, DNA oligo with phosphorothioate linker; 2'-OMe-PS, 2'-*O*-methyl chemistry with phosphorothioate linker; 2'-MOE-PS, 2'-*O*-methoxyethyl chemistry with phosphorothioate linker; PMO, phosphorodiamidate morpholino oligomers.

both fall under the MedDRA term "nausea and vomiting symptoms". This could relate to two different patients, one experiencing nausea and one experiencing vomiting, or one patient experiencing both nausea and vomiting (which should not be registered twice). The minimum count included 12,622 total reported AEs while the maximum count included 14,566 total reported AEs. All analyses in this meta-analysis were performed using the minimum AE count. To test for the impact of this potential bias, we estimated the pooled incidences for the top 20 most-reported AEs for both minimum and maximum AE count datasets. For all 20 events, there was no significant difference in incidence between the minimum and the maximum counts (Figure S1), indicating that the conservative minimum counts are a valid dataset from which to draw conclusions.

Confounding factors in meta-analysis can be addressed through meta-regression

Apart from any bias in counting methods that results from differences in reporting, there are also large differences between studies in both ASO target and design. These differences between clinical studies serve as confounding factors, leading to increased measured heterogeneity (I^2).[123] To address heterogeneity, we implemented a meta-regression to control for several of these confounding factors. These analyses provide insight into the observed heterogeneity that results from the differences between studies.[124]

Meta-regression was performed in order to analyze whether an AE was caused by ASO treatment or another confounding factor. Each study was assigned to a group for every study variable that could serve as a confounding factor. These variables included ASO chemistry, ASO administration route, ASO mode of action, patient disease or diagnosis, patient age, whether the study was placebo controlled, and study publication year (Table S4). This list of study variables is not exhaustive, however, since relative variables such as dosage, treatment time, and follow-up time are difficult to compare between studies. GalNAc conjugation was also considered as a regression variable, but this did not lead to any meaningful data due to too small sample size. Apart from the study variables that could not be included, other undiscovered confounding factors may also be involved. Furthermore, the absence of significant differences of study variables on the effect sizes in the meta-regression analyses does not indicate that the AE effect size is only caused by the ASO treatment. Rather, it indicates that the heterogeneity that was observed between studies cannot be explained (entirely) by the confounding factors that were tested for, which can point toward a significant effect of ASO treatment or the effect of an undiscovered confounding factor. Nevertheless, these meta-regression analyses contribute to a deeper understanding of the impact of ASOs on the occurrence of AEs.

Incidence analysis on a large ASO adverse event dataset reveals common adverse event resulting from ASO treatment

As an overall first analysis, the complete dataset of 101 articles was pooled and used in an incidence analysis of AEs. Incidence

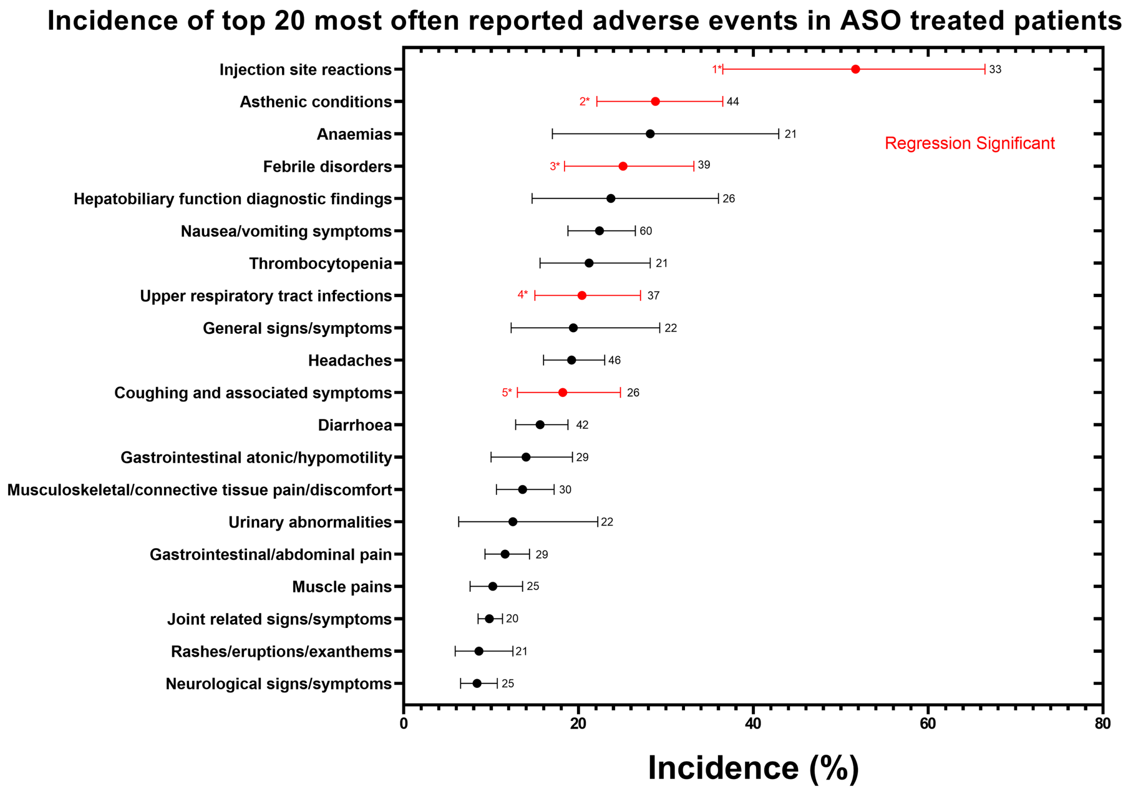

Figure 2. Incidence of top 20 most most-reported adverse events in ASO-treated patients

Shown are the pooled incidences of AEs (0%-100%, 95% confidence intervals) in the studies reporting on the respective AEs. Alongside each point is the number of studies that have reported on this adverse event. Data points shown in red indicate a significant outcome was observed in their respective regression analysis. 1*: significant impact on effect size in regression analysis due to PMO chemistry, 2*: significant impact due to a patient population consisting of cancer patients, 3*: significant impact due to intrathecal injection, 4*: significant impact due to a patient population consisting of muscle degenerative disease patients, and 5*: significant impact due to intrathecal injection.

analysis measures how often a certain AE occurs in the study population, but these event rates are not placebo-controlled. In this analysis "injection site reactions" were reported in 33 studies with a 51.7% incidence rate and a 36.5%–66.5% confidence interval of 95%. "Nausea and vomiting symptoms" are the most-often reported AEs across studies (60 studies, 22.4%: 18.8%–26.5%). Other frequently reported AEs are general events such as "headaches" (46 studies, 19.2%: 16.0%–23.0%), "fevers" (labeled with the MedDRA term "febrile disorders" in 39 studies, 25.1%: 18.4%–33.2%) and "fatigue/asthenic conditions" (44 studies, 28.8%: 22.1%–36.5%). AEs of note include "anemia" (21 studies, 28.2%: 17.0%–42.9%), and liver issues that fall under the MedDRA term "hepatobiliary function diagnostics procedures," which includes events like deregulated or increased/decreased liver enzymes, alanine transaminase or aspartate transferase, bilirubin, or gamma-glutamyl transferase (26 studies, 23.7%: 14.7%–36.0%). Figure 2 lists the incidences of the 20 most-reported AEs in ASO-treated patients. These data provide insights into the incidence of AEs that occur in clinical studies with ASOs. All AE incidence rates that were reported in three or more studies are shown in Table S2.

Risk difference analysis provides insight into the increased risk of adverse events resulting from ASO treatment in placebo-controlled trials

Next to assessing the overall incidence over the whole dataset, a risk difference analysis was performed. The risk difference analysis shows the AEs that have a statistically significant increased risk compared with placebo. Between 47 placebo-controlled studies, 8 AEs had a significantly increased risk. Figure 3 shows the increased risk of AEs in ASO-treated patients compared with placebo-treated patients. Injection site reactions were the highest risk AE at 37.6% increased risk, with a 95% confidence interval of 27.9%–47.2%. The most often reported AE was "nausea and vomiting symptoms," which was reported in 18 studies (6.5%: 10.7%–21.3%).

Only 2 of the 47 placebo-controlled studies directly reported thrombocytopenia (11.1%: 5.1%–17.1%), which was found more frequently in ASO-treated groups compared with placebo-treated groups. Anemias were also reported in 3 studies with an increased risk difference (7.0%: 0.8%–13.2%). No meta-regression analysis findings were reported for any of the significantly increased AEs. Other significantly increased AEs included feelings and sensations (such as chills or

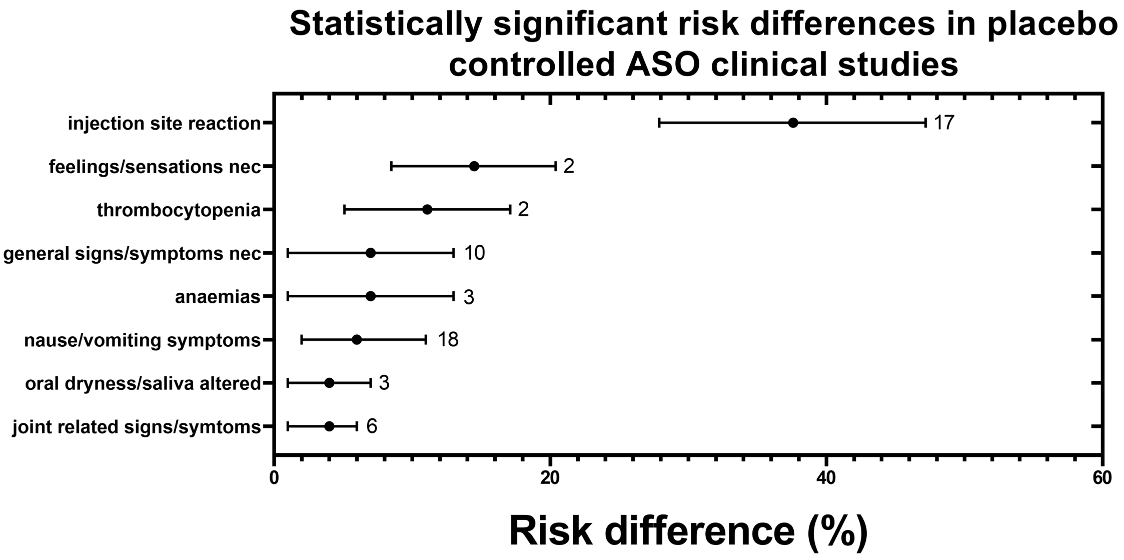

Figure 3. Statistically significant risk differences between ASO-treated and placebo-treated patients
Results are presented as percentage increase in AE risk of ASO treatment over placebo with the respective 95% upper and lower confidence intervals. Beside each point is the number of studies that have reported on this AE. No significant impact on the effect sizes presented here was observed in the meta-regression analyses, implying that the variables tested for in the regression analyses did not significantly impact the observed effects sizes.

shivers), general signs and symptoms, oral dryness/altered saliva, and joint-related signs and symptoms.

Subgroup analysis reveals the similarities and differences between adverse event resulting from separate ASO design characteristics

Next, we performed separate meta-analyses on subgroups to study the potential effects of different design properties; administration route, ASO chemistry, and mode of action on AE rates. Figure 4 shows that studies using intravenous administration presented significantly higher incidences than subcutaneous administration for all significant effects (anemias, vascular hypotensive disorders, lower respiratory tract infection, asthenic conditions, and nausea/vomiting), except for injection site reactions. Analysis between ASO chemistries did not yield any significant differences in AE incidence rates, except for injection site reactions, where the pooled incidence is significantly higher for the 2’-MOE and 2’-*O*Me chemistry compared with the PMO chemistry (2’-MOE chemistry: 18 studies, 58.9%: 38.7%–76.5% and 2’*O*Me chemistry: 7 studies, 75.8%: 49.3%–90.9% compared with the PMO chemistry: 4 studies, 8.7%: 3.9%–18.0%).

We also observed a significant difference in coagulation and bleeding incidence by mode of action, with a significantly higher AE incidence in RNase-H mode-of-action studies compared with splice modification studies (Splice modification: 4 studies, 5.3%: 1.9%–13.8%; RNase H: 7 studies, 52.7%: 31.4%–73.1%) (Table S1).

The 10 most frequently reported AEs per ASO design property are shown in Table 3, including the most common design specifications from a chemistry, administration, and mode-of-action perspective. Headaches are the only AE to feature in the top 10 across all categories. Injection site reactions consistently have the highest incidence rate across the different categories. For the remaining variables where injection site reactions did not make the list, incidences are 8.7% (3.9%–18.0%) over 4 studies for PMO, 12.5% (8.1%–18.9%) over 7 studies for intravenous infusion, and 36.0% (16.0%–62.5%) over 11 studies for splice-altering ASOs. Injection site reactions were only mentioned once in intrathecal studies. The intravenous administration category presented the most serious AEs, including comparatively high incidences of AEs such as liver symptoms, thrombocytopenia, and anemia.

DISCUSSION

This systematic review provides a comprehensive overview of the available literature on AEs in ASO-treated patients. Our meta-analysis summarizes AE incidences and risk differences over a large dataset of 101 studies in which human patients received an ASO therapy, including drugs never submitted or approved for market authorization. This meta-analysis provides safety information on AEs in ASO treatments that are not specifically tied to one specific disease or drug. The main purpose of our study was to provide quantitative estimates of AE rates resulting from ASO treatments. With N-of-1 ASO therapies,[5] in which regular clinical trials cannot be performed, previously untreated patients with rare diseases could be treated with experimental therapies with an uncertain safety profile. The meta-analysis data from this study can thus serve as a base for a general ASO safety profile for use when setting up such N-of-1 trials.

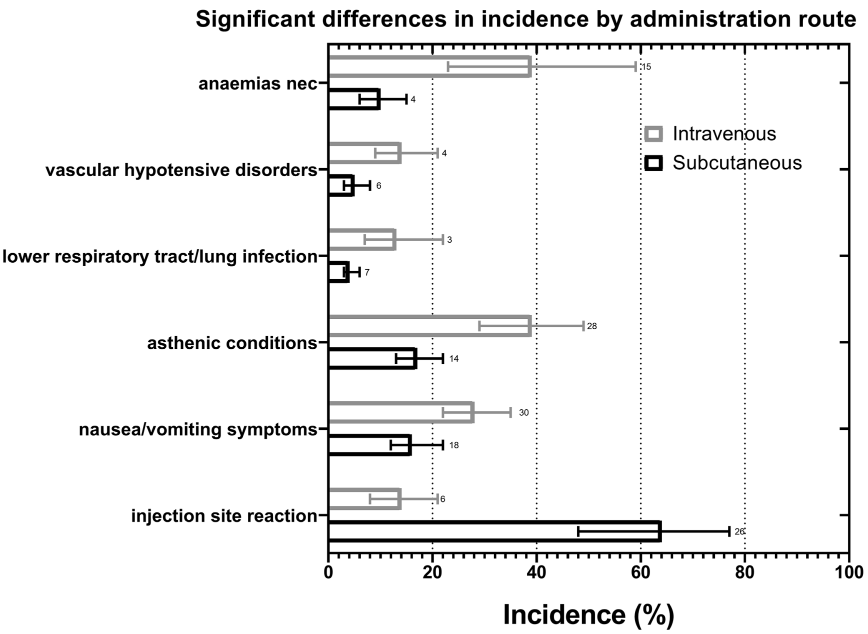

Figure 4. Significant differences in AE incidence between intravenous and subcutaneous administration

NEC is a MedDRA term for "not elsewhere clarified". No significant effects of regression variables were found.

(Figure S2). Several studies described coagulation-related events as "lowered platelet counts" or "bleeding analysis diagnostic findings," among other terms. These terms can be considered related but not identical to thrombocytopenia and fall under MedDRA terms other than "thrombocytopenia". These terms, therefore, could not be included in the analysis of thrombocytopenia. When we did merge these events with thrombocytopenia, the overall pooled risk difference of thrombocytopenia was no longer significantly different. Figure S2 provides this sub-analysis with merged terms, showing a very small but significantly increased risk difference between ASO-treated and placebo-treated groups for 2'-MOE-PS ASO chemistry but not for 2'-*O*Me-PS chemistry, which explains the non-significant difference observed in the overall analysis. Heterogeneity in these separate subgroups is also a bit lower than in the overall analysis, suggesting that heterogeneity in overall effect size could partially be the result of between-study variability in chemistry. These findings are in high contrast to anecdotal evidence[127] and underscore underreporting of AEs in studies in humans.

Interpreting the results obtained from the meta-analysis requires addressing the limiting factors inherent to this study. This dataset includes a variety of compounds including early/first generation ASOs and next-generation ligand-conjugated ASOs within the same analyses. These ASOs differ substantially in dosing requirements, tissue distribution, and individual compound safety profiles, and any analysis that attempts to include these within the same dataset must account for these differences. Several limitations have been addressed through our analysis methods, such as zero-count correction, minimum versus maximum count correction, and meta-regression. However, as described earlier, the list of variables included in the meta-regression is not exhaustive, and undiscovered confounding factors could still influence the final result.

The results obtained in this meta-analysis demonstrate how the pooling of clinical data can contribute to a data-driven general safety profile of ASOs. The known sequence-independent AEs were also reflected in our meta-analysis, where we found high incidences for renal function analyses 12.2% (7.2%–19.8%) (Table S2) and hepatobiliary function and diagnostic findings 23.7% (14.7%–36.0%) (subcategory 1), febrile disorders 25.1% (18.4%–33.2%) (subcategory 2), injection site reactions 49.8% (34.9%–64.8%) (subcategory 3), and thrombocytopenia 21.2% (15.6%–28.2%) (subcategory 4). General symptoms such as fatigue, fever, nausea and vomiting, and headaches are also commonly reported for ASO treatments, irrespective of ASO design.

Thrombocytopenia is considered a common AE of ASO treatment and is often attributed to the PS backbone modification of the ASO.[125,126] In the PMO selection, there were no reports of thrombocytopenia. Interestingly, we observed a difference in the incidence of thrombocytopenia-related events between MOE and *O*Me chemistry

While AEs such as thrombocytopenia and liver or renal symptoms can be found across all types of studies, studies using intravenous administration seem to present the most severe safety profile, with relatively increased incidence and reporting frequency of thrombocytopenia, anemia, and hepatobiliary function diagnostic events. This may be partially explained by the difference in pharmacokinetics between administration routes. For instance, peak plasma concentration is reached faster and is higher after intravenous administration compared with subcutaneous administration,[128] which can influence ASO concentrations in tissue and, consequently, may affect AE rates.

The general perception in literature on ASO design is that PMO chemistry is chosen in order to achieve a lower risk of AEs compared with other chemistries.[129] However, the PMO chemistry can only be used as a splice alteration oligo, since the PMO chemistry does not support the RNase-H mechanism of action. Our results also suggest that PMO chemistry has a milder safety profile[130–132] compared with 2'-*O*Me-PS and 2'-MOE-PS,[133] with comparatively lower AE incidence rates and fewer severe AEs. An example of note is injection site reactions, which are absent in the list of most-reported events for the PMO chemistry (Table 3). This result likely stems from the fact that ten out of eleven trials administering PMOs do so

Table 3. Top 10 of most reported AEs per ASO design variable

2’-*O*Me-PS	Incidence (95% CI)	Studies	2’-MOE-PS	Incidence (95% CI)	Studies	PMO[a]	Incidence (95% CI)	Studies
Injection site reaction	75.8 (49.3-90.9)	7	injection site reaction	58.9 (38.7-76.5)	18	upper respiratory tract infection	34.2 (18.4-54.5)	7
Asthenic conditions	49.9 (21.5-78.3)	6	general signs/symptoms	20.9 (11.2-35.6)	16	coughing/associated symptoms	30.0 (18.0-45.5)	6
Urinary abnormalities	45.0 (6.2-91.2)	5	upper respiratory tract infection	20.8 (13.5-28.9)	21	headaches	27.9 (18.5-39.7)	8
Nausea/vomiting symptoms	25.4 (13.6-42.4)	11	asthenic conditions	19.6 (14.8-25.4)	14	nausea/vomiting symptoms	25.2 (13.8-41.5)	7
Febrile disorders	24.9 (14.9-38.6)	8	headaches	19.3 (14.4-25.3)	22	musculoskeletal/connective tissue pain/discomfort	23.6 (15.9-33.6)	7
Hepatobiliary function diagnostics	22.7 (0.2-81.0)	5	nausea/vomiting symptoms	18.7 (14.5-23.9)	23	urinary abnormalities	16.1 (3.8-48.2)	6
Coughing/associated symptoms	18.4 (14.0-23.8)	5	diarrhea excluding infective	17.0 (12.9-22.1)	15	febrile disorders	16.0 (7.6-30.5)	7
Diarrhea excluding infective	18.3 (10.1-30.9)	7	coughing/associated symptoms	16.1 (9.2-26.7)	13	dermatitis/eczema	12.9 (6.9-22.8)	7
Headaches	17.8 (11.7-26.2)	6	musculoskeletal/connective tissue pain/discomfort	13.8 (10.4-18.3)	17	rashes/eruption/exanthems	10.5 (3.8-26.0)	6
Gastrointestinal/ abdominal pain	9.6 (6.3-14.1)	5	muscle pains	11.5 (7.9-16.5)	12	diarrhea excluding infective	10.4 (5.6-18.4)	6
**Intrathecal**	**Incidence (95% CI)**	**Studies**	**Intravenous**	**Incidence (95% CI)**	**Studies**	**Subcutaneous**	**Incidence (95% CI)**	**Studies**
Febrile disorders	59.0 (32.0-81.4)	6	anemias	39.3 (22.9-58.6)	15	injection site reaction	63.9 (48.4-76.9)	26
Coughing/associated symptoms	36.0 (19.0-57.5)	6	asthenic conditions	39.2 (29.9-49.4)	28	general signs/symptoms	24.1 (13.9-38.6)	15
Upper respiratory tract infection	30.2 (12.5-56.7)	8	hepatobiliary function diagnostics	28.9 (18.1-42.6)	14	headaches	18.7 (14.3-24.0)	21
Headaches	27.5 (17.4-40.6)	5	nausea/vomiting symptoms	29.0 (23.2-35.6)	30	upper respiratory tract infection	17.9 (12.6-24.8)	19
Diarrhea excluding infective	23.6(15.2-34.9)	6	febrile disorders	27.8 (19.4-38.0)	23	asthenic conditions	16.9 (12.8-22.0)	14
Lower respiratory tract/lung infection	22.8 (8.0-50.1)	15	thrombocytopenia	23.3 (15.6-33.3)	16	nausea/vomiting symptoms	16.3 (12.1-21.6)	18
Nausea/vomiting symptoms	22.5 (13.4-35.3)	9	appetite disorders	21.9 (12.4-35.7)	13	diarrhea excluding infective	14.7 (10.7-19.9)	14
Non-site-specific injuries	22.5 (13.2-35.7)	6	headaches	20.4 (15.1-27.0)	17	muscle pains	11.1 (7.5-16.2)	13
Musculoskeletal/connective tissue pain/discomfort	19.4 (13.1-27.8)	7	gastrointestinal/ abdominal pain	15.3 (13.0-18.0)	14	gastrointestinal/abdominal pain	10.6 (7.2-15.3)	12
Gastrointestinal/abdominal pain	17.4 (5.3-44.4)	6	diarrhea excluding infective	15.7 (11.8-20.6)	23	musculoskeletal/connective tissue pain/discomfort	9.9 (7.5-13.0)	16

Splice alteration	Incidence (95% CI)	Studies	RNase H	Incidence (95% CI)	Studies
Upper respiratory tract infection	32.1 (21.2-45.4)	17	injection site reaction	59.0 (40.5-75.2)	22
Febrile disorders	29.5 (17.3-45.6)	16	asthenic conditions[b]	30.4 (23.4-38.4)	38
Coughing/associated symptoms	29.1 (19.8-40.4)	14	hepatobiliary function diagnostics	28.6 (18.1-42.1)	22
Headaches	26.9 (21.6-32.9)	15	febrile disorders	25.8 (17.3-36.7)	21
Urinary abnormalities	23.9 (8.1-52.6)	12	nausea/vomiting symptoms	22.6 (18.2-27.7)	40
Nausea/vomiting symptoms	21.8 (15.2-30.1)	19	headaches	17.1 (13.6-21.4)	31

(*Continued on next page*)

Table 3. *Continued*

Splice alteration	Incidence (95% CI)	Studies	RNase H	Incidence (95% CI)	Studies
Diarrhea excluding infective	16.6 (12.4-22.0)	14	diarrhea excluding infective	16.4 (12.9-20.7)	27
Musculoskeletal/connective tissue pain/ discomfort	16.5 (11.5-23.3)	16	upper respiratory tract infection	15.1 (11.1-20.1)	20
Dermatitis/eczema	14.3 (9.8-20.5)	13	gastrointestinal/abdominal pain	11.8 (9.1-15.2)	21
Rashes/eruption/exanthems	9.6 (4.8-18.2)	12	muscle pains	10.2 (7.4-14.0)	21

Effect size is incidence in percentage with 95% confidence intervals (95% CI). Events are sorted based on incidence. Abbreviations: DNA-PS, DNA oligo with phosphorothioate linker; 2’-OMe-PS, 2’-*O*-methyl chemistry with phosphorothioate linker; 2’-MOE-PS, 2’-*O*-methoxyethyl chemistry with phosphorothioate linker; PMO, phosphorodiamidate morpholino oligomers.
[a]Regression analysis could not be performed due to collinearity of the regression variables for the PMO chemistry.
[b]Significant impact on asthenic conditions effect size due to cancer patients in regression analysis.

intravenously. Because of the similarity in these PMO trials, the data extracted from PMO studies was highly collinear. This means that a proper meta-regression could not be performed, since these studies largely targeted the same pathology and used the same administration route and target population. Furthermore, there is less AE data on PMO-treated patients available than that on patients treated with other chemistries (Table 3). The PMO chemistry is different in that the neutral charge of the molecule requires higher dosages to achieve similar biodistribution compared with the charged PS backbone ASOs,[7] which may introduce other potential AEs that are not yet well understood.[8] In contrast, 2’-*O*Me-PS and 2’-MOE-PS chemistries have been more extensively tested.

Since the date of our original search, 25 additional studies have been published that fit our inclusion criteria.[134–158] As they could not be retroactively added into the dataset, we qualitatively screened these articles based on their treated patient and AE counts in a post hoc analysis. Interestingly, the recent articles reported more frequently on thrombocytopenia compared with the original set of articles, showing that this AE has gained more attention in more recent literature. We found that 5 out of 25 articles mentioned thrombocytopenia specifically, while 10 mentioned platelet reductions or reported on the absence of platelet-specific AEs.

To compare the data of these new studies with the original dataset, we compared the incidences of the top 20 most frequently occurring AEs with those in the post hoc articles. In these studies, the AE counts generally fell within the confidence intervals of AEs observed in our meta-analysis (Figure 2). For example, in the study by Yuen and colleagues, which had the largest population and reported a trial on bepirovirsen, a 2’MOE-PS ASO targeting Hepatitis B ,[158] the incidences of injection site reactions (62.6% incidence) and hepatobiliary findings (19.5% incidence) were not different from the incidences reported in our meta-analysis. One exception was thrombocytopenia. Four articles provided quantifiable data on thrombocytopenia, which resulted in a slightly higher incidence (0.4% increase for a 28.6% total) compared with our original findings. This difference is caused by the aforementioned study on bepirovirsen where 80 out of 229 patients were reported with thrombocytopenia. No new AEs were reported in these more recent articles. Common AE reports in these articles included headaches, injection site reactions, general symptoms, upper respiratory tract infections, and diarrhea, again with incidences not different from our meta-analysis.

Recommendations

Better availability on safety data in a diverse and expanding scientific field could propel the rate at which new developments take place. Recommendations based on the findings of this study are summarized in Table 4. The analysis in this review was limited by missing data and heterogeneous reporting, largely due to underreporting or inconsistent registration methods. Not all AEs could be studied in full detail. Several studies reported that event rates were only listed for AEs that occurred in >10% of the study population. In meta-analysis, incomplete reporting leads to false negatives and systematic underreporting, manifested here as increased uncertainty. Studies that used cut-off measurements or other methods of data collection and registration that could lead to bias have been marked in the risk of bias analysis (Table S3). For example, only 8 of the 47 placebo-controlled studies reported symptoms related to coagulation events. Event counts in the placebo groups of these eight studies suggested that these symptoms also appeared in the placebo-treated study population. However, in the remaining 40 studies, similar events were neither reported in the ASO-treated nor in the placebo-treated populations. A possible explanation could be the selective reporting if the event only occurs in >10% of the population. Another explanation could be that these symptoms or events were simply not measured or recorded, and therefore, not detected or reported. The same situation is true for other AEs, leading to limited data availability for meta-analysis, despite the inclusion of 101 studies. To address this issue in the future, more attention could be paid to ASO-related specific reporting.[14] One example of such a recommended way of reporting was found in the post hoc literature search.[149] Oral and colleagues included a separate table reporting platelet reduction counts within the study population even when these did not lead to thrombocytopenia.[149] Brannagan and colleagues mentioned the number of missed doses due to platelet monitoring safety.[135] These are good examples of considering the known effects of ASOs in combination with classical AE reporting, which would not specifically report such events. With regards to registration, AEs resulting from ASO treatment

Table 4. Summary of recommendations for increasing the availability of safety data in the field of antisense oligonucleotides

Recommendation	Rationale	Expected impact
Zero registration	registering known ASO-specific events as zero would increase the confidence of subsequent data analysis and remove doubt as to whether an event did not take place or was missed in testing or not tested for.	more safety data availability improved safety profile for ASOs due to registration of non-occurrences.
Avoiding cut-off thresholds	cut-off thresholds decreases detection rates for rare events, as event reporting is dependent on the size of the study population.	more safety data availability improved safety profile for ASOs due to registration of non-occurrences.
ASO-specific monitoring	ASO-related events such as decreased platelets may require a laboratory test in order to be detected; standardizing such a test would increase the detection rate.	increased detection rate of ASO specific events
Increased collaboration	ASOs are usually targeted at smaller groups of patients leading to low study populations; by increasingly cooperating and sharing safety data, a general safety profile for ASOs may become more defined.	stimulate new developments of ASOs

should not be subject to cut-off values for measurement. In the context of the small trials inherent to ASOs for rare disease, this leads to imply that rare AEs are not being reported or published. Such an initiative would enable better pooling of AE data across studies, thereby ensuring more reliable safety profiles for ASOs. This would also allow for better assessment of the impact of ASO chemistry and administration routes on AEs and provide sufficient data to understand the underlying causes of common ASO-related AEs. Data resulting from a common ASO AE screening and reporting strategy will be useful for both researchers developing ASOs and clinicians aiming to treat a specific disease.

The ASO field is very diverse and rapidly developing. ASOs are undergoing improvements, and there are multiple ongoing clinical trials with newly developed ASOs. ASOs are also increasingly being developed as a personalized therapy for N-of-1 cases. While this variety in disorders and targets complicates the standardization of ASO development procedures, there are ongoing collaborations aimed at establishing frameworks to facilitate ASO development,[11] for example, in the case of N-of-1 ASOs, the European 1M1M collaboration, n-Lorem, and the global N=1 Collaborative.[159] Through increased collaboration facilitated by these initiatives between researchers and research groups, knowledge about safety data and a route through the regulatory system may be more readily shared, benefitting new ASO developments.

Concluding remarks

Reporting on AEs in clinical studies is not consistent across studies due to, for example, different cut-off values used for reporting an AE. This lack of consistent reporting and measurement of ASO-induced AEs, combined with small and heterogeneous study populations treated with heterogeneous compounds, creates substantial challenges in assessing safety data. Altogether, this underscores the critical knowledge gap that hampers clinical translation of new (N = 1) ASO therapies. This meta-analysis offers a novel overview of AEs across many different types of ASOs in an analytical fashion. Our review provides perspectives on AEs in ASO treatments that should be considered when designing ASOs and clinical studies. Increased and standardized reporting of AEs resulting from ASO therapies will lead to increased understanding of ASO-specific safety profiles, which will help shape regulations and streamline both research into ASO development and clinical trial design.

MATERIALS AND METHODS

A systematic review (PROSPERO ID: CRD42023345231) and meta-analysis was designed and conducted in accordance with the Cochrane handbook for systematic reviews and interventions[20] and the PRISMA 2020 guidelines checklist.[160]

Literature search

A specialized term block was used to capture any variation in the language used to describe AEs (i.e., toxicity or side effect) (supplemental information). The search strategy was validated in PubMed and adapted for Embase, Cochrane, and Web of Science search engines. Retrieved articles were checked for duplicates and subsequently assessed through their title and abstract for inclusion. Articles that were included based on title and abstract were assessed in a full text screening. From the full text screening, articles were included in the dataset if they treated humans with ASOs (no combination therapy) and published quantified AE data in tables. Both the title/abstract and full text screenings were performed *in duplo* by two authors (C.V. and J.B.) independently using Rayyan,[161] and disagreements were resolved by discussion between the authors.

Extraction

Data extraction was performed by retrieving the raw AE data and the following study characteristics: date of publication, types of ASOs used, ASO chemistry, administration route, and patient cohort information such as age, patient diagnosis, treatment duration, and follow-up time. Classification data was obtained from the full text of the article. For example, if the study mentioned an MOE ASO, it is reported this way in the dataset. Due to differences in reporting methodology among studies, AE reports were harmonized using MedDRA terminology. Software package *R* (R Foundation for Statistical Computing, Vienna, Austria) was used to sort and filter the data.

Risk of bias

Risk of bias was assessed for each included study according to the Cochrane risk of bias guidelines. After assessment by two authors (C.V. and R.R.V.), discrepancies in assessments were resolved

through unblinded discussion. Types of bias assessed included performance bias, detection bias, attrition bias, reporting bias, information bias, and confounding bias (Table S3).[20]

Analysis

To carry out meta-analyses and meta-regression analyses, extracted and sorted data was imported to Stata 18 (StataCorp. 2023. Stata Statistical Software: Release 18. College Station, TX: StataCorp LLC). Two main types of meta-analysis were performed. First, AE incidence over all 101 articles was pooled with a logit-transformed proportion effect size using a random effect restricted maximum likelihood meta-analysis (REML) model. The second analysis was a binary outcome analysis investigating the pooled risk difference in the 48 placebo-controlled studies (REML model). Data on AEs from studies using specific ASO design properties were used to compare AE incidence rates between these ASO properties. This could not be performed on the risk difference data due to insufficient data availability (low or no AE counts) in placebo-controlled clinical studies.

Meta-analysis was also performed on sub-groups, calculating effect size differences between chemistries, administration routes, and mode of action. We performed sub-analyses using the REML model on either incidence or risk difference data. Data were depicted using Stata or GraphPad Prism (GraphPad Software, Boston, MA, USA). A sensitivity analysis was also performed in order to account for potential biases in extracted (minimum and maximum) event counts.

Meta-regression

Meta-regression analysis was performed to account for heterogeneity (I^2) across studies that can confound the true effect size of AE rates. For each AE in each analysis, the heterogeneity of pooled AE incidences and risk differences was quantified using a multivariable meta-regression analysis (REML model). Meta-regression provides information on the effect size of study-specific variables and their significance level on pooled event rates. The following study-specific variables were included for analysis: ASO chemistry, ASO administration route, ASO mode of action, patient disease or diagnosis, patient age, whether the study was placebo-controlled, and study publication year (Table S4). Age was categorized as low (0−18 years), medium (19−60 years), or high (65+ y). Year of publication was categorized as early (1990−2010), medium (2011–2017), or late (2018−2023), based on innovations in the ASO field.[162–164] Constructed: “early” articles concern the earliest trials and first-approved ASOs, “medium” articles reflect developments in a time when more ASOs were being developed and approved, and “late” articles reflect the latest developments, usually for new and untested drugs or modifications still undergoing trials. If the regression result for a variable is significant, between-study heterogeneity can be at least partially explained by that factor. Consequently, the study factor in question is significantly associated with the effect size found in the meta-analysis, and the true impact of the ASO treatment on the AEs observed may, therefore, be different than the meta-analysis result.

Meta-regression was performed separately for each analysis and each variable except for the PMO chemistry regression (Table 3) due to collinearity between the studies reporting trials of PMOs. For example, 10 of the 11 studies that used PMOs administered ASO intravenously, and 10 of these 11 studies treated patients with Duchenne muscular dystrophy. A further 9 out of 11 studies reported treating patients under the age of 18 only.

Zero correction

In studies that do not report on a certain AE, it is often unclear whether this reflects absence of events or absence of reporting. In the case of absence of data due to reporting this would suggest false negative data. In a maximum likelihood analysis, zero events can lead to issues in the statistical analyses. To avoid having to apply zero-cell[124,165] correction to a large group of studies, we only used studies that reported on a specific event in question included in the analysis.

ACKNOWLEDGMENTS

The authors would like to thank Kate McIntyre for her assistance with grammar and language editing in this manuscript. This work was sponsored by the Dutch Butterfly Child Foundation (Stichting Vlinderkind) (research grant to P.C.v.d.A., J.B., and M.C.B.) and Dioraphte (research grant to J.B.).

AUTHOR CONTRIBUTIONS

Conceptualization, C.V., J.B., and P.C.v.d.A; review design, C.V., J.B., P.C.v.d.A., and M. C.B.; literature screening, C.V. and J.B.; data extraction and organization, C.V.; statistical analysis, C.V. and E.B.; risk of bias analysis, C.V. and R.R.V., original draft, C.V.; review and editing, all authors; project oversight, J.B. and P.C.v.d.A.; funding acquisition, J.B. and P.C.v.d.A. All authors have read and agreed to the published version of the manuscript.

DECLARATION OF INTERESTS

The authors declare no conflict of interest.

SUPPLEMENTAL INFORMATION

Supplemental information can be found online at https://doi.org/10.1016/j.omtn.2026.102976.

REFERENCES

1. Lauffer, M.C., van Roon-Mom, W., and Aartsma-Rus, A.; N. Collaborative (2024). Possibilities and limitations of antisense oligonucleotide therapies for the treatment of monogenic disorders. Commun Med (Lond). *4*, 6.
2. Dhuri, K., Bechtold, C., Quijano, E., Pham, H., Gupta, A., Vikram, A., and Bahal, R. (2020). Antisense Oligonucleotides: An Emerging Area in Drug Discovery and Development. J. Clin. Med. *9*, 2004.
3. Crooke, S.T. (2017). Molecular Mechanisms of Antisense Oligonucleotides. Nucleic Acid Ther. *27*, 70–77.
4. Aartsma-Rus, A., and van Ommen, G.J.B. (2007). Antisense-mediated exon skipping: a versatile tool with therapeutic and research applications. RNA *13*, 1609–1624.
5. Kim, J., Hu, C., Moufawad El Achkar, C., Black, L.E., Douville, J., Larson, A., Pendergast, M.K., Goldkind, S.F., Lee, E.A., Kuniholm, A., et al. (2019). Patient-Customized Oligonucleotide Therapy for a Rare Genetic Disease. N. Engl. J. Med. *381*, 1644–1652.
6. Quemener, A.M., Bachelot, L., Forestier, A., Donnou-Fournet, E., Gilot, D., and Galibert, M.D. (2020). The powerful world of antisense oligonucleotides: From bench to bedside. Wiley Interdiscip. Rev. RNA *11*, e1594.
7. Lu, Q.L., Yokota, T., Takeda, S., Garcia, L., Muntoni, F., and Partridge, T. (2011). The status of exon skipping as a therapeutic approach to duchenne muscular dystrophy. Mol. Ther. *19*, 9–15.

8. Sabrina Haque, U., Kohut, M., and Yokota, T. (2024). Comprehensive review of adverse reactions and toxicology in ASO-based therapies for Duchenne Muscular Dystrophy: From FDA-approved drugs to peptide-conjugated ASO. Curr. Res. Toxicol. *7*, 100182.
9. Debacker, A.J., Voutila, J., Catley, M., Blakey, D., and Habib, N. (2020). Delivery of Oligonucleotides to the Liver with GalNAc: From Research to Registered Therapeutic Drug. Mol. Ther. *28*, 1759–1771.
10. Ruan, H., Dou, D., Lu, J., Xiao, X., Gong, X., and Zhang, X. (2025). Off-target effects of oligonucleotides and approaches of preclinical assessments. SLAS Discov. *35*, 100254.
11. Aartsma-Rus, A., Garanto, A., van Roon-Mom, W., McConnell, E.M., Suslovitch, V., Yan, W.X., Watts, J.K., and Yu, T.W. (2023). Consensus Guidelines for the Design and In Vitro Preclinical Efficacy Testing N-of-1 Exon Skipping Antisense Oligonucleotides. Nucleic Acid Ther. *33*, 17–25.
12. Frazier, K.S. (2015). Antisense oligonucleotide therapies: the promise and the challenges from a toxicologic pathologist's perspective. Toxicol. Pathol. *43*, 78–89.
13. Collotta, D., Bertocchi, I., Chiapello, E., and Collino, M. (2023). Antisense oligonucleotides: a novel Frontier in pharmacological strategy. Front. Pharmacol. *14*, 1304342.
14. Goyenvalle, A., Jimenez-Mallebrera, C., van Roon, W., Sewing, S., Krieg, A.M., Arechavala-Gomeza, V., and Andersson, P. (2023). Considerations in the Preclinical Assessment of the Safety of Antisense Oligonucleotides. Nucleic Acid Ther. *33*, 1–16.
15. Andersson, P. (2022). Preclinical Safety Assessment of Therapeutic Oligonucleotides. Methods Mol. Biol. *2434*, 355–370.
16. Slingsby, M.H.L., Vijey, P., Tsai, I.T., Roweth, H., Couldwell, G., Wilkie, A.R., Gaus, H., Goolsby, J.M., Okazaki, R., Terkovich, B.E., et al. (2022). Sequence-specific 2'-O-methoxyethyl antisense oligonucleotides activate human platelets through glycoprotein VI, triggering formation of platelet-leukocyte aggregates. Haematologica *107*, 519–531.
17. Valenzuela, A., Ayuso, M., Buyssens, L., Bars, C., Van Ginneken, C., Tessier, Y., and Van Cruchten, S. (2023). Platelet Activation by Antisense Oligonucleotides (ASOs) in the Gottingen Minipig, including an Evaluation of Glycoprotein VI (GPVI) and Platelet Factor 4 (PF4) Ontogeny. Pharmaceutics *15*.
18. Alhamadani, F., Zhang, K., Parikh, R., Wu, H., Rasmussen, T.P., Bahal, R., Zhong, X.B., and Manautou, J.E. (2022). Adverse Drug Reactions and Toxicity of the Food and Drug Administration-Approved Antisense Oligonucleotide Drugs. Drug Metab. Dispos. *50*, 879–887.
19. Fletcher, J. (2006). What is heterogeneity and is it important? BMJ *333*, 83–86.
20. Chandler, J., Cumpston, M., Li, T., Page, M.J., and Welch, V.A. (2024). Cochrane Handbook for Systematic Reviews of Interventions version. In Version 6.5, J. Higgins and J. Thomas, eds. (Cochrane).
21. Ackermann, E.J., Guo, S., Benson, M.D., Booten, S., Freier, S., Hughes, S.G., Kim, T. W., Jesse Kwoh, T., Matson, J., Norris, D., et al. (2016). Suppressing transthyretin production in mice, monkeys and humans using 2nd-Generation antisense oligonucleotides. Amyloid. *23*, 148–157.
22. Acsadi, G., Crawford, T.O., Müller-Felber, W., Shieh, P.B., Richardson, R., Natarajan, N., Castro, D., Ramirez-Schrempp, D., Gambino, G., Sun, P., et al. (2021). Safety and efficacy of nusinersen in spinal muscular atrophy: The EMBRACE study. Muscle Nerve *63*, 668–677.
23. Advani, R., Lum, B.L., Fisher, G.A., Halsey, J., Geary, R.S., Holmlund, J.T., Kwoh, T. J., Dorr, F.A., and Sikic, B.I. (2005). A phase I trial of aprinocarsen (ISIS 3521/LY900003), an antisense inhibitor of protein kinase C-alpha administered as a 24-hour weekly infusion schedule in patients with advanced cancer. Invest. N. Drugs *23*, 467–477.
24. Advani, R., Peethambaram, P., Lum, B.L., Fisher, G.A., Hartmann, L., Long, H.J., Halsey, J., Holmlund, J.T., Dorr, A., and Sikic, B.I. (2004). A Phase II trial of aprinocarsen, an antisense oligonucleotide inhibitor of protein kinase C alpha, administered as a 21-day infusion to patients with advanced ovarian carcinoma. Cancer *100*, 321–326.
25. Akdim, F., Tribble, D.L., Flaim, J.D., Yu, R., Su, J., Geary, R.S., Baker, B.F., Fuhr, R., Wedel, M.K., and Kastelein, J.J.P. (2011). Efficacy of apolipoprotein B synthesis inhibition in subjects with mild-to-moderate hyperlipidaemia. Eur. Heart J. *32*, 2650–2659.
26. Bayever, E., Iversen, P.L., Bishop, M.R., Sharp, J.G., Tewary, H.K., Arneson, M.A., Pirruccello, S.J., Ruddon, R.W., Kessinger, A., Zon, G., et al. (1993). Systemic administration of a phosphorothioate oligonucleotide with a sequence complementary to p53 for acute myelogenous leukemia and myelodysplastic syndrome: initial results of a phase I trial. Antisense Res. Dev. *3*, 383–390.
27. Beigel, J.H., Voell, J., Muñoz, P., Kumar, P., Brooks, K.M., Zhang, J., Iversen, P., Heald, A., Wong, M., and Davey, R.T. (2018). Safety, tolerability, and pharmacokinetics of radavirsen (AVI-7100), an antisense oligonucleotide targeting influenza a M1/M2 translation. Br. J. Clin. Pharmacol. *84*, 25–34.
28. Benson, M.D., Waddington-Cruz, M., Berk, J.L., Polydefkis, M., Dyck, P.J., Wang, A.K., Planté-Bordeneuve, V., Barroso, F.A., Merlini, G., Obici, L., et al. (2018). Inotersen treatment for patients with Hereditary transthyretin amyloidosis. N. Engl. J. Med. Overseas. Ed. *379*, 22–31.
29. Bianchini, D., Omlin, A., Pezaro, C., Lorente, D., Ferraldeschi, R., Mukherji, D., Crespo, M., Figueiredo, I., Miranda, S., Riisnaes, R., et al. (2013). First-in-human Phase i study of EZN-4176, a locked nucleic acid antisense oligonucleotide to exon 4 of the androgen receptor mRNA in patients with castration-resistant prostate cancer. Br. J. Cancer *109*, 2579–2586.
30. Chen, H.X., Marshall, J.L., Ness, E., Martin, R.R., Dvorchik, B., Rizvi, N., Marquis, J., McKinlay, M., Dahut, W., and Hawkins, M.J. (2000). A safety and pharmacokinetic study of a mixed-backbone oligonucleotide (GEM231) targeting the type I protein kinase A by two-hour infusions in patients with refractory solid tumors. Clin. Cancer Res. *6*, 1259–1266.
31. Chi, K.N., Yu, E.Y., Jacobs, C., Bazov, J., Kollmannsberger, C., Higano, C.S., Mukherjee, S.D., Gleave, M.E., Stewart, P.S., and Hotte, S.J. (2016). A phase I dose-escalation study of apatorsen (OGX-427), an antisense inhibitor targeting heat shock protein 27 (Hsp27), in patients with castration-resistant prostate cancer and other advanced cancers. Ann. Oncol. *27*, 1116–1122.
32. Chiriboga, C.A., Swoboda, K.J., Darras, B.T., Iannaccone, S.T., Montes, J., De Vivo, D.C., Norris, D.A., Bennett, C.F., and Bishop, K.M. (2016). Results from a phase 1 study of nusinersen (ISIS-SMN(Rx)) in children with spinal muscular atrophy. Neurology *86*, 890–897.
33. Cideciyan, A.V., Jacobson, S.G., Drack, A.V., Ho, A.C., Charng, J., Garafalo, A.V., Roman, A.J., Sumaroka, A., Han, I.C., Hochstedler, M.D., et al. (2019). Effect of an intravitreal antisense oligonucleotide on vision in Leber congenital amaurosis due to a photoreceptor cilium defect. Nat. Med. *25*, 225–228.
34. Cirak, S., Arechavala-Gomeza, V., Guglieri, M., Feng, L., Torelli, S., Anthony, K., Abbs, S., Garralda, M.E., Bourke, J., Wells, D.J., et al. (2011). Exon skipping and dystrophin restoration in patients with Duchenne muscular dystrophy after systemic phosphorodiamidate morpholino oligomer treatment: an open-label, phase 2, dose-escalation study. Lancet *378*, 595–605.
35. Clemens, P.R., Rao, V.K., Connolly, A.M., Harper, A.D., Mah, J.K., McDonald, C. M., Smith, E.C., Zaidman, C.M., Nakagawa, T., Hoffman, E.P., et al. (2022). Long-Term Functional Efficacy and Safety of Viltolarsen in Patients with Duchenne Muscular Dystrophy. J. Neuromuscul. Dis. *9*, 493–501.
36. Clemens, P.R., Rao, V.K., Connolly, A.M., Harper, A.D., Mah, J.K., Smith, E.C., McDonald, C.M., Zaidman, C.M., Morgenroth, L.P., Osaki, H., et al. (2020). Safety, Tolerability, and Efficacy of Viltolarsen in Boys With Duchenne Muscular Dystrophy Amenable to Exon 53 Skipping A Phase 2 Randomized Clinical Trial. JAMA Neurol. *77*, 982–991.
37. Coudert, B., Anthoney, A., Fiedler, W., Droz, J.P., Dieras, V., Borner, M., Smyth, J. F., Morant, R., De Vries, M.J., and RoelvinkP. Fumoleau, M.; European Organization for Research and Treatment of Cancer EORTC (2001). Phase II trial with ISIS 5132 in patients with small-cell (SCLC) and non-small cell (NSCLC) lung cancer. A European Organization for Research and Treatment of Cancer (EORTC) early clinical studies group report. Eur. J. Cancer *37*, 2194–2198.
38. Cripps, M.C., Figueredo, A.T., Oza, A.M., Taylor, M.J., Fields, A.L., Holmlund, J.T., McIntosh, L.W., Geary, R.S., and Eisenhauer, E.A. (2002). Phase II randomized study of ISIS 3521 and ISIS 5132 in patients with locally advanced or metastatic colorectal cancer: A National Cancer Institute of Canada clinical trials group study. Clin. Cancer Res. *8*, 2188–2192.
39. Cunningham, C.C., Holmlund, J.T., Schiller, J.H., Geary, R.S., Kwoh, T.J., Dorr, A., and Nemunaitis, J. (2000). A phase I trial of c-raf kinase antisense oligonucleotide

ISIS 5132 administered as a continuous intravenous infusion in patients with advanced cancer. Clin. Cancer Res. *6*, 1626–1631.

40. Davis, A.J., Gelmon, K.A., Siu, L.L., Moore, M.J., Britten, C.D., Mistry, N., Klamut, H., D'Aloisio, S., MacLean, M., Wainman, N., et al. (2003). Phase I and pharmacologic study of the human DNA methyltransferase antisense oligodeoxynucleotide MG98 given as a 21-day continuous infusion every 4 weeks. Invest. N. Drugs *21*, 85–97.

41. De Vivo, D.C., Bertini, E., Swoboda, K.J., Hwu, W.L., Crawford, T.O., Finkel, R.S., Kirschner, J., Kuntz, N.L., Parsons, J.A., Ryan, M.M., et al. (2019). Nusinersen initiated in infants during the presymptomatic stage of spinal muscular atrophy: Interim efficacy and safety results from the Phase 2 NURTURE study. Neuromuscul. Disord. *29*, 842–856.

42. Desai, A.A., Schilsky, R.L., Young, A., Janisch, L., Stadler, W.M., Vogelzang, N.J., Cadden, S., Wright, J.A., and Ratain, M.J. (2005). A phase I study of antisense oligonucleotide GTI-2040 given by continuous intravenous infusion in patients with advanced solid tumors. Ann. Oncol. *16*, 958–965.

43. Drevinek, P., Pressler, T., Cipolli, M., De Boeck, K., Schwarz, C., Bouisset, F., Boff, M., Henig, N., Paquette-Lamontagne, N., Montgomery, S., et al. (2020). Antisense oligonucleotide eluforsen is safe and improves respiratory symptoms in F508DEL cystic fibrosis. J. Cyst. Fibros. *19*, 99–107.

44. Fijen, L.M., Riedl, M.A., Bordone, L., Bernstein, J.A., Raasch, J., Tachdjian, R., Craig, T., Lumry, W.R., Manning, M.E., Alex, V.J., and Cohn, D.M. (2022). Inhibition of Prekallikrein for Hereditary Angioedema. N. Engl. J. Med. Overseas. Ed. *386*, 1026–1033.

45. Finkel, R.S., Chiriboga, C.A., Vajsar, J., Day, J.W., Montes, J., De Vivo, D.C., Bishop, K.M., Foster, R., Liu, Y., Ramirez-Schrempp, D., et al. (2021). Treatment of infantile-onset spinal muscular atrophy with nusinersen: final report of a phase 2, open-label, multicentre, dose-escalation study. Lancet Child Adolesc. Health *5*, 491–500.

46. Finkel, R.S., Mercuri, E., Darras, B.T., Connolly, A.M., Kuntz, N.L., Kirschner, J., Chiriboga, C.A., Saito, K., Servais, L., Tizzano, E., et al.; ENDEAR Study Group (2017). Nusinersen versus Sham Control in Infantile-Onset Spinal Muscular Atrophy. N. Engl. J. Med. *377*, 1723–1732.

47. Flaim, J.D., Grundy, J.S., Baker, B.F., McGowan, M.P., and Kastelein, J.J.P. (2014). Changes in mipomersen dosing regimen provide similar exposure with improved tolerability in randomized placebo-controlled study of healthy volunteers. J. Am. Heart Assoc. *3*, e000560.

48. Flanigan, K.M., Voit, T., Rosales, X.Q., Servais, L., Kraus, J.E., Wardell, C., Morgan, A., Dorricott, S., Nakielny, J., Quarcoo, N., et al. (2014). Pharmacokinetics and safety of single doses of drisapersen in non-ambulant subjects with Duchenne muscular dystrophy: Results of a double-blind randomized clinical trial. Neuromuscul. Disord. *24*, 16–24.

49. Gane, E., Yuen, M.F., Kim, D.J., Chan, H.L.Y., Surujbally, B., Pavlovic, V., Das, S., Triyatni, M., Kazma, R., Grippo, J.F., et al. (2021). Clinical Study of Single-Stranded Oligonucleotide RO7062931 in Healthy Volunteers and Patients With Chronic Hepatitis B. Hepatology *74*, 1795–1808.

50. Gaudet, D., Alexander, V.J., Baker, B.F., Brisson, D., Tremblay, K., Singleton, W., Geary, R.S., Hughes, S.G., Viney, N.J., Graham, M.J., et al. (2015). Antisense Inhibition of Apolipoprotein C-III in Patients with Hypertriglyceridemia. N. Engl. J. Med. Overseas. Ed. *373*, 438–447.

51. Goel, S., Desai, K., Bulgaru, A., Fields, A., Goldberg, G., Agrawal, S., Martin, R., Grindel, M., and Mani, S. (2003). A safety study of a mixed-backbone oligonucleotide (GEM231) targeting the type I regulatory subunit α of protein kinase A using a continuous infusion schedule in patients with refractory solid tumors. Clin. Cancer Res. *9*, 4069–4076.

52. Goemans, N., Mercuri, E., Belousova, E., Komaki, H., Dubrovsky, A., McDonald, C. M., Kraus, J.E., Lourbakos, A., Lin, Z., Campion, G., et al.; DEMAND III study groupI (2018). A randomized placebo-controlled phase 3 trial of an antisense oligonucleotide, drisapersen, in Duchenne muscular dystrophy. Neuromuscul. Disord. *28*, 4–15.

53. Goemans, N.M., Tulinius, M., Van Den Akker, J.T., Burm, B.E., Ekhart, P.F., Heuvelmans, N., Holling, T., Janson, A.A., Platenburg, G.J., Sipkens, J.A., et al. (2011). Systemic administration of PRO051 in Duchenne's muscular dystrophy. N. Engl. J. Med. *364*, 1513–1522.

54. Goemans, N.M., Tulinius, M., van den Hauwe, M., Kroksmark, A.K., Buyse, G., Wilson, R.J., van Deutekom, J.C., de Kimpe, S.J., Lourbakos, A., and Campion, G. (2016). Long-Term Efficacy, Safety, and Pharmacokinetics of Drisapersen in Duchenne Muscular Dystrophy: Results from an Open-Label Extension Study. PLoS One *11*, e0161955.

55. Gouni-Berthold, I., Alexander, V.J., Yang, Q.Q., Hurh, E., Steinhagen-Thiessen, E., Moriarty, P.M., Hughes, S.G., Gaudet, D., Hegele, R.A., Witztum, J.L., et al. (2021). Efficacy and safety of volanesorsen in patients with multifactorial chylomicronaemia (COMPASS): a multicentre, double-blind, randomised, placebo-controlled, phase 3 trial. Lancet Diabetes Endocrinol. *9*, 264–275.

56. Hagenacker, T., Wurster, C.D., Günther, R., Schreiber-Katz, O., Osmanovic, A., Petri, S., Weiler, M., Ziegler, A., Kuttler, J., Koch, J.C., et al. (2020). Nusinersen in adults with 5q spinal muscular atrophy: a non-interventional, multicentre, observational cohort study. Lancet Neurol. *19*, 317–325.

57. Han, K., Cremer, J., Elston, R., Oliver, S., Baptiste-Brown, S., Chen, S., Gardiner, D., Davies, M., Saunders, J., Hamatake, R., et al. (2019). A Randomized, Double-Blind, Placebo-Controlled, First-Time-in-Human Study to Assess the Safety, Tolerability, and Pharmacokinetics of Single and Multiple Ascending Doses of GSK3389404 in Healthy Subjects. Clin. Pharmacol. Drug Dev. *8*, 790–801.

58. Hong, D.S., Kurzrock, R., Oh, Y., Wheler, J., Naing, A., Brail, L., Callies, S., André, V., Kadam, S.K., Nasir, A., et al. (2011). A phase 1 dose escalation, pharmacokinetic, and pharmacodynamic evaluation of eIF-4E antisense oligonucleotide LY2275796 in patients with advanced cancer. Clin. Cancer Res. *17*, 6582–6591.

59. Jachimczak, P., Schlingensiepen, K.H., Heinrichs, H., and Bogdahn, U. (2010). Targeted therapy of high-grade gliomas using TGF-beta2 inhibitor trabedersen (AP 12009): results of the Phase IIb study as basis for the Phase III SAPPHIRE Study. Cancer Res. *70*.

60. Jeong, W., Rapisarda, A., Park, S.R., Kinders, R.J., Chen, A., Melillo, G., Turkbey, B., Steinberg, S.M., Choyke, P., Doroshow, J.H., et al. (2014). Pilot trial of EZN-2968, an antisense oligonucleotide inhibitor of hypoxia-inducible factor-1 alpha (HIF-1α), in patients with refractory solid tumors. Cancer Chemother. Pharmacol. *73*, 343–348.

61. Kain, H., Goldblum, D., Geudelin, B., Thorin, E., and Beglinger, C. (2009). Tolerability and safety of GS-101 eye drops, an antisense oligonucleotide to insulin receptor substrate-1: A 'first in man' Phase i investigation. Br. J. Clin. Pharmacol. *68*, 169–173.

62. Kastelein, J.J.P., Wedel, M.K., Baker, B.F., Su, J., Bradley, J.D., Yu, R.Z., Chuang, E., Graham, M.J., and Crooke, R.M. (2006). Potent reduction of apolipoprotein B and low-density lipoprotein cholesterol by short-term administration of an antisense inhibitor of apolipoprotein B. Circulation *114*, 1729–1735.

63. Klisovic, R.B., Stock, W., Cataland, S., Klisovic, M.I., Liu, S., Blum, W., Green, M., Odenike, O., Godley, L., Marcucci, G., et al. (2008). A phase I biological study of MG98, an oligodeoxynucleotide antisense to DNA methyltransferase 1, in patients with high-risk myelodysplasia and acute myeloid leukemia. Clin. Cancer Res. *14*, 2444–2449.

64. Komaki, H., Nagata, T., Saito, T., Masuda, S., Takeshita, E., Sasaki, M., Tachimori, H., Nakamura, H., Aoki, Y., and Takeda, S. (2018). Systemic administration of the antisense oligonucleotide NS-065/NCNP-01 for skipping of exon 53 in patients with Duchenne muscular dystrophy. Sci. Transl. Med. *10*, eaan0713.

65. Komaki, H., Takeshima, Y., Matsumura, T., Ozasa, S., Funato, M., Takeshita, E., Iwata, Y., Yajima, H., Egawa, Y., Toramoto, T., et al. (2020). Viltolarsen in Japanese Duchenne muscular dystrophy patients: A phase 1/2 study. Ann. Clin. Transl. Neurol. *7*, 2393–2408.

66. Levine, A.M., Tulpule, A., Quinn, D.I., Gorospe, G., 3rd, Smith, D.L., Hornor, L., Boswell, W.D., Espina, B.M., Groshen, S.G., Masood, R., et al. (2006). Phase I study of antisense oligonucleotide against vascular endothelial growth factor: Decrease in plasma vascular endothelial growth factor with potential clinical efficacy. J. Clin. Oncol. *24*, 1712–1719.

67. Loomba, R., Morgan, E., Watts, L., Xia, S., Hannan, L.A., Geary, R.S., Baker, B.F., and Bhanot, S. (2020). Novel antisense inhibition of diacylglycerol O-acyltransferase 2 for treatment of non-alcoholic fatty liver disease: a multicentre, double-blind, randomised, placebo-controlled phase 2 trial. Lancet Gastroenterol. Hepatol. *5*, 829–838.

68. Maksymowych, W.P., Blackburn, W.D., Tami, J.A., and Shanahan, W.R. (2002). A randomized, placebo controlled trial of an antisense oligodeoxynucleotide to

intercellular adhesion molecule-1 in the treatment of severe rheumatoid arthritis. J. Rheumatol. *29*, 447–453.

69. Marshall, J.L., Eisenberg, S.G., Johnson, M.D., Hanfelt, J., Dorr, F.A., El-Ashry, D., Oberst, M., Fuxman, Y., Holmlund, J., and Malik, S. (2004). A phase II trial of ISIS 3521 in patients with metastatic colorectal cancer. Clin. Colorectal Cancer *4*, 268–274.

70. McDonald, C.M., Shieh, P.B., Abdel-Hamid, H.Z., Connolly, A.M., Ciafaloni, E., Wagner, K.R., Goemans, N., Mercuri, E., Khan, N., Koenig, E., et al. (2021). Open-Label Evaluation of Eteplirsen in Patients with Duchenne Muscular Dystrophy Amenable to Exon 51 Skipping: PROMOVI Trial. J. Neuromuscul. Dis. *8*, 989–1001.

71. McDonald, C.M., Wong, B., Flanigan, K.M., Wilson, R., de Kimpe, S., Lourbakos, A., Lin, Z., Campion, G.; the DEMAND V study group, and Study, V. (2018). Placebo-controlled Phase 2 Trial of Drisapersen for Duchenne Muscular Dystrophy. Ann. Clin. Transl. Neurol. *5*, 913–926.

72. McGowan, M.P., Tardif, J.C., Ceska, R., Burgess, L.J., Soran, H., Gouni-Berthold, I., Wagener, G., and Chasan-Taber, S. (2012). Randomized, placebo-controlled trial of mipomersen in patients with severe hypercholesterolemia receiving maximally tolerated lipid-lowering therapy. PLoS One *7*, e49006.

73. Mendell, J.R., Goemans, N., Lowes, L.P., Alfano, L.N., Berry, K., Shao, J., Kaye, E. M., and Mercuri, E. (2016). Longitudinal effect of eteplirsen versus historical control on ambulation in Duchenne muscular dystrophy. Ann. Neurol. *79*, 257–271.

74. Mendell, J.R., Rodino-Klapac, L.R., Sahenk, Z., Roush, K., Bird, L., Lowes, L.P., Alfano, L., Gomez, A.M., Lewis, S., Kota, J., et al. (2013). Eteplirsen for the treatment of Duchenne muscular dystrophy. Ann. Neurol. *74*, 637–647.

75. Mercuri, E., Darras, B.T., Chiriboga, C.A., Day, J.W., Campbell, C., Connolly, A.M., Iannaccone, S.T., Kirschner, J., Kuntz, N.L., Saito, K., et al.; CHERISH Study Group (2018). Nusinersen versus sham control in later-onset spinal muscular atrophy. N. Engl. J. Med. *378*, 625–635.

76. Miller, T., Cudkowicz, M., Shaw, P.J., Andersen, P.M., Atassi, N., Bucelli, R.C., Genge, A., Glass, J., Ladha, S., Ludolph, A.L., et al. (2020). Phase 1-2 Trial of antisense oligonucleotide tofersen for SOD1 ALS. N. Engl. J. Med. Overseas. Ed. *383*, 109–119.

77. Miller, T.M., Pestronk, A., David, W., Rothstein, J., Simpson, E., Appel, S.H., Andres, P.L., Mahoney, K., Allred, P., Alex, K., et al. (2013). An antisense oligonucleotide against SOD1 delivered intrathecally for patients with SOD1 familial amyotrophic lateral sclerosis: A phase 1, randomised, first-in-man study. Lancet Neurol. *12*, 435–442.

78. Monteleone, G., Fantini, M.C., Onali, S., Zorzi, F., Sancesario, G., Bernardini, S., Calabrese, E., Viti, F., Monteleone, I., Biancone, L., et al. (2012). Phase i clinical trial of smad7 knockdown using antisense oligonucleotide in patients with active crohn's disease. Mol. Ther. *20*, 870–876.

79. Morgan, E., Tami, Y., Hu, K., Mullick, A., Geary, R., and Tsimikas, S. (2021). EFFECT OF IONIS-AGT-LRX, AN ANTISENSE INHIBITOR OF ANGIOTENSINOGEN PRODUCTION, IN HEALTHY VOLUNTEERS AND IN SUBJECTS WITH HYPERTENSION: RESULTS OF PHASE 1 AND PHASE 2 STUDIES. J. Am. Coll. Cardiol. *77*, 1549.

80. Morris, M.J., Tong, W.P., Cordon-Cardo, C., Drobnjak, M., Kelly, W.K., Slovin, S. F., Terry, K.L., Siedlecki, K., Swanson, P., Rafi, M., et al. (2002). Phase I trial of BCL-2 antisense oligonucleotide (G3139) administered by continuous intravenous infusion in patients with advanced cancer. Clin. Cancer Res. *8*, 679–683.

81. Nemunaitis, J., Holmlund, J.T., Kraynak, M., Richards, D., Bruce, J., Ognoskie, N., Kwoh, T.J., Geary, R., Dorr, A., Von Hoff, D., et al. (1999). Phase I evaluation of ISIS 3521, an antisense oligodeoxynucleotide to protein kinase C-alpha, in patients with advanced cancer. J. Clin. Oncol. *17*, 3586–3595.

82. Noveck, R., Stroes, E.S.G., Flaim, J.D., Baker, B.F., Hughes, S., Graham, M.J., Crooke, R.M., and Ridker, P.M. (2014). Effects of an antisense oligonucleotide inhibitor of C-reactive protein synthesis on the endotoxin challenge response in healthy human male volunteers. J. Am. Heart Assoc. *3*, e001084.

83. O'Brien, S.M., Cunningham, C.C., Golenkov, A.K., Turkina, A.G., Novick, S.C., and Rai, K.R. (2005). Phase I to II multicenter study of oblimersen sodium, a Bcl-2 antisense oligonucleotide, in patients with advanced chronic lymphocytic leukemia. J. Clin. Oncol. *23*, 7697–7702.

84. Oza, A.M., Elit, L., Swenerton, K., Faught, W., Ghatage, P., Carey, M., McIntosh, L., Dorr, A., Holmlund, J.T., and Eisenhauer, E. (2003). Phase II study of CGP 69846A (ISIS 5132) in recurrent epithelial ovarian cancer: An NCIC clinical trials group study (NCIC IND.116). Gynecol. Oncol. *89*, 129–133.

85. Pfeiffer, N., Voykov, B., Renieri, G., Bell, K., Richter, P., Weigel, M., Thieme, H., Wilhelm, B., Lorenz, K., Feindor, M., et al. (2017). First-in-human phase I study of ISTH0036, an antisense oligonucleotide selectively targeting transforming growth factor beta 2 (TGF-beta 2),in subjects with open-angle glaucoma undergoing glaucoma filtration surgery. PLoS One *12*, e0188899.

86. Plummer, R., Vidal, L., Griffin, M., Lesley, M., De Bono, J., Coulthard, S., Sludden, J., Siu, L.L., Chen, E.X., Oza, A.M., et al. (2009). Phase I study of MG98, an oligonucleotide antisense inhibitor of human DNA methyltransferase 1, given as a 7-day infusion in patients with advanced solid tumors. Clin. Cancer Res. *15*, 3177–3183.

87. Raal, F.J., Braamskamp, M.J., Selvey, S.L., Sensinger, C.H., and Kastelein, J.J. (2016). Pediatric experience with mipomersen as adjunctive therapy for homozygous familial hypercholesterolemia. J. Clin. Lipidol. *10*, 860–869.

88. Raal, F.J., Santos, R.D., Blom, D.J., Marais, A.D., Charng, M.J., Cromwell, W.C., Lachmann, R.H., Gaudet, D., Tan, J.L., Chasan-Taber, S., et al. (2010). Mipomersen, an apolipoprotein B synthesis inhibitor, for lowering of LDL cholesterol concentrations in patients with homozygous familial hypercholesterolaemia: a randomised, double-blind, placebo-controlled trial. Lancet *375*, 998–1006.

89. Dean, E., Jodrell, D., Connolly, K., Danson, S., Jolivet, J., Durkin, J., Morris, S., Jowle, D., Ward, T., Cummings, J., et al. (2009). Phase I trial of AEG35156 administered as a 7-day and 3-day continuous intravenous infusion in patients with advanced refractory cancer. J. Clin. Oncol. *27*, 1660–1666.

90. Rao, S., Watkins, D., Cunningham, D., Dunlop, D., Johnson, P., Selby, P., Hancock, B.W., Fegan, C., Culligan, D., Schey, S., et al. (2004). Phase II study of ISIS 3521, an antisense oligodeoxynucleotide to protein kinase C alpha, in patients with previously treated low-grade non-Hodgkin's lymphoma. Ann. Oncol. *15*, 1413–1418.

91. Reeskamp, L.F., Kastelein, J.J.P., Moriarty, P.M., Duell, P.B., Catapano, A.L., Santos, R.D., and Ballantyne, C.M. (2019). Safety and efficacy of mipomersen in patients with heterozygous familial hypercholesterolemia. Atherosclerosis *280*, 109–117.

92. Reilley, M.J., McCoon, P., Cook, C., Lyne, P., Kurzrock, R., Kim, Y., Woessner, R., Younes, A., Nemunaitis, J., Fowler, N., et al. (2018). STAT3 antisense oligonucleotide AZD9150 in a subset of patients with heavily pretreated lymphoma: results of a phase 1b trial. J. Immunother. Cancer *6*, 119.

93. Roqué, F., Mon, G., Belardi, J., Rodriguez, A., Grinfeld, L., Long, R., Grossman, S., Malcolm, A., Zon, G., Ormont, M.L., et al. (2001). Safety of intracoronary administration of c-myc antisense oligomers after percutaneous transluminal coronary angioplasty (PTCA). Antisense Nucleic Acid Drug Dev. *11*, 99–106.

94. Rudin, C.M., Holmlund, J., Fleming, G.F., Mani, S., Stadler, W.M., Schumm, P., Monia, B.P., Johnston, J.F., Geary, R., Yu, R.Z., et al. (2001). Phase I trial of ISIS 5132, an antisense oligonucleotide inhibitor of c-raf-1, administered by 24-hour weekly infusion to patients with advanced cancer. Clin. Cancer Res. *7*, 1214–1220.

95. Rudin, C.M., Marshall, J.L., Huang, C.H., Kindler, H.L., Zhang, C., Kumar, D., Gokhale, P.C., Steinberg, J., Wanaski, S., Kasid, U.N., et al. (2004). Delivery of a liposomal c-raf-1 antisense oligonucleotide by weekly bolus dosing in patients with advanced solid tumors: A phase I study. Clin. Cancer Res. *10*, 7244–7251.

96. Russell, S.R., Drack, A.V., Cideciyan, A.V., Jacobson, S.G., Leroy, B.P., Van Cauwenbergh, C., Ho, A.C., Dumitrescu, A.V., Han, I.C., Martin, M., et al. (2022). Intravitreal antisense oligonucleotide sepofarsen in Leber congenital amaurosis type 10: a phase 1b/2 trial. Nat. Med. *28*, 1014–1021.

97. Sands, B.E., Feagan, B.G., Sandborn, W.J., Schreiber, S., Peyrin-Biroulet, L., Frédéric Colombel, J., Rossiter, G., Usiskin, K., Ather, S., Zhan, X., et al. (2020). Mongersen (GED-0301) for Active Crohn's Disease: results of a Phase 3 Study. Am. J. Gastroenterol. *115*, 738–745.

98. Santos, R.D., Duell, P.B., East, C., Guyton, J.R., Moriarty, P.M., Chin, W., and Mittleman, R.S. (2015). Long-term efficacy and safety of mipomersen in patients with familial hypercholesterolaemia: 2-year interim results of an open-label extension. Eur. Heart J. *36*, 566–575.

99. Sermet-Gaudelus, I., Clancy, J.P., Nichols, D.P., Nick, J.A., De Boeck, K., Solomon, G.M., Mall, M.A., Bolognese, J., Bouisset, F., den Hollander, W., et al. (2019).

Antisense oligonucleotide eluforsen improves CFTR function in F508del cystic fibrosis. J. Cyst. Fibros. *18*, 536–542.

100. Servais, L., Mercuri, E., Straub, V., Guglieri, M., Seferian, A.M., Scoto, M., Leone, D., Koenig, E., Khan, N., Dugar, A., et al. (2022). Long-Term Safety and Efficacy Data of Golodirsen in Ambulatory Patients with Duchenne Muscular Dystrophy Amenable to Exon 53 Skipping: A First-in-human, Multicenter, Two-Part, Open-Label, Phase 1/2 Trial. Nucleic Acid Therapeut. *32*, 29–39.

101. Stein, E.A., Dufour, R., Gagne, C., Gaudet, D., East, C., Donovan, J.M., Chin, W., Tribble, D.L., and McGowan, M. (2012). Apolipoprotein B Synthesis Inhibition With Mipomersen in Heterozygous Familial Hypercholesterolemia. Circulation *126*, 2283–2292.

102. Stevenson, J.P., Yao, K.S., Gallagher, M., Friedland, D., Mitchell, E.P., Cassella, A., Monia, B., Kwoh, T.J., Yu, R., Holmlund, J., et al. (1999). Phase I clinical pharmacokinetic and pharmacodynamic trial of the c-raf-1 antisense oligonucleotide ISIS 5132 (CGP 69846A). J. Clin. Oncol. *17*, 2227–2236.

103. Stewart, D.J., Donehower, R.C., Eisenhauer, E.A., Wainman, N., Shah, A.K., Bonfils, C., MacLeod, A.R., Besterman, J.M., and Reid, G.K. (2003). A phase I pharmacokinetic and pharmacodynamic study of the DNA methyltransferase 1 inhibitor MG98 administered twice weekly. Ann. Oncol. *14*, 766–774.

104. Tanioka, M., Nokihara, H., Yamamoto, N., Yamada, Y., Yamada, K., Goto, Y., Fujimoto, T., Sekiguchi, R., Uenaka, K., Callies, S., et al. (2011). Phase i study of LY2181308, an antisense oligonucleotide against survivin, in patients with advanced solid tumors. Cancer Chemother. Pharmacol. *68*, 505–511.

105. Tardif, J.C., Karwatowska-Prokopczuk, E., Amour, E.S., Ballantyne, C.M., Shapiro, M.D., Moriarty, P.M., Baum, S.J., Hurh, E., Bartlett, V.J., Kingsbury, J., et al. (2022). Apolipoprotein C-III reduction in subjects with moderate hypertriglyceridaemia and at high cardiovascular risk. Eur. Heart J. *43*, 1401–1412.

106. Täubel, J., Hauke, W., Rump, S., Viereck, J., Batkai, S., Poetzsch, J., Rode, L., Weigt, H., Genschel, C., Lorch, U., et al. (2021). Novel antisense therapy targeting microRNA-132 in patients with heart failure: Results of a first-in-human Phase 1b randomized, double-blind, placebo-controlled study. Eur. Heart J. *42*, 178–188.

107. Thomas, G.S., Cromwell, W.C., Ali, S., Chin, W., Flaim, J.D., and Davidson, M. (2013). Mipomersen, an Apolipoprotein B Synthesis Inhibitor, Reduces Atherogenic Lipoproteins in Patients With Severe Hypercholesterolemia at High Cardiovascular Risk. J. Am. Coll. Cardiol. *62*, 2178–2184.

108. Trainer, P.J., Newell-Price, J.D.C., Ayuk, J., Aylwin, S.J.B., Rees, A., Drake, W., Chanson, P., Brue, T., Webb, S.M., Fajardo, C., et al. (2018). A randomised, open-label, parallel group phase 2 study of antisense oligonucleotide therapy in acromegaly. Eur. J. Endocrinol. *179*, 97–108.

109. Van Meer, L., Moerl, M., Van Dongen, M., Goulouze, B., De Kam, M., Klaassen, E., Cohen, A., and Burggraa, J. (2016). Renal effects of antisense-mediated inhibition of SGLT2. J. Pharmacol. Exp. Therapeut. *359*, 280–289.

110. Viney, N.J., Guo, S., Tai, L.J., Baker, B.F., Aghajan, M., Jung, S.W., Yu, R.Z., Booten, S., Murray, H., Machemer, T., et al. (2021). Ligand conjugated antisense oligonucleotide for the treatment of transthyretin amyloidosis: preclinical and phase 1 data. ESC Heart Fail. *8*, 652–661.

111. Visser, M.E., Akdim, F., Tribble, D.L., Nederveen, A.J., Kwoh, T.J., Kastelein, J.J.P., Trip, M.D., and Stroes, E.S.G. (2010). Effect of apolipoprotein-B synthesis inhibition on liver triglyceride content in patients with familial hypercholesterolemia. JLR (J. Lipid Res.) *51*, 1057–1062.

112. Visser, M.E., Wagener, G., Baker, B.F., Geary, R.S., Donovan, J.M., Beuers, U.H.W., Nederveen, A.J., Verheij, J., Trip, M.D., Basart, D.C.G., et al. (2012). Mipomersen, an apolipoprotein B synthesis inhibitor, lowers low-density lipoprotein cholesterol in high-risk statin-intolerant patients: A randomized, double-blind, placebo-controlled trial. Eur. Heart J. *33*, 1142–1149.

113. Voit, T., Topaloglu, H., Straub, V., Muntoni, F., Deconinck, N., Campion, G., De Kimpe, S.J., Eagle, M., Guglieri, M., Hood, S., et al. (2014). Safety and efficacy of drisapersen for the treatment of Duchenne muscular dystrophy (DEMAND II): an exploratory, randomised, placebo-controlled phase 2 study. Lancet Neurol. *13*, 987–996.

114. Wagner, K.R., Kuntz, N.L., Koenig, E., East, L., Upadhyay, S., Han, B., and Shieh, P. B. (2021). Safety, tolerability, and pharmacokinetics of casimersen in patients with Duchenne muscular dystrophy amenable to exon 45 skipping: A randomized, double-blind, placebo-controlled, dose-titration trial. Muscle Nerve *64*, 285–292.

115. Warren, M.S., Hughes, S.G., Singleton, W., Yamashita, M., and Genovese, M.C. (2015). Results of a proof of concept, double-blind, randomized trial of a second generation antisense oligonucleotide targeting high-sensitivity C-reactive protein (hs-CRP) in rheumatoid arthritis. Arthritis Res. Ther. *17*, 80.

116. Waters, J.S., Webb, A., Cunningham, D., Clarke, P.A., Raynaud, F., di Stefano, F., and Cotter, F.E. (2000). Phase I clinical and pharmacokinetic study of bcl-2 antisense oligonucleotide therapy in patients with non-Hodgkin's lymphoma. J. Clin. Oncol. *18*, 1812–1823.

117. Winquist, E., Knox, J., Ayoub, J.P., Wood, L., Wainman, N., Reid, G.K., Pearce, L., Shah, A., and Eisenhauer, E. (2006). Phase II trial of DNA methyltransferase 1 inhibition with the antisense oligonucleotide MG98 in patients with metastatic renal carcinoma: A National Cancer Institute of Canada Clinical Trials Group investigational new drug study. Invest. N. Drugs *24*, 159–167.

118. Witztum, J.L., Gaudet, D., Freedman, S.D., Alex, V.J., Digenio, A., Williams, K.R., Yang, Q., Hughes, S.G., Geary, R.S., Bruckert, E., et al. (2019). Volanesorsen and triglyceride levels in familial chylomicronemia syndrome. N. Engl. J. Med. Overseas. Ed. *381*, 531–542.

119. Yacyshyn, B., Chey, W.Y., Wedel, M.K., Yu, R.Z., Paul, D., and Chuang, E. (2007). A Randomized, Double-Masked, Placebo-Controlled Study of Alicaforsen, an Antisense Inhibitor of Intercellular Adhesion Molecule 1, for the Treatment of Subjects With Active Crohn's Disease. Clin. Gastroenterol. Hepatol. *5*, 215–220.

120. Yacyshyn, B.R., Chey, W.Y., Goff, J., Salzberg, B., Baerg, R., Buchman, A.L., Tami, J., Yu, R., Gibiansky, E., and Shanahan, W.R.; ISIS 2302-CS9 Investigators (2002). Double blind, placebo controlled trial of the remission inducing and steroid sparing properties of an ICAM-1 antisense oligodeoxynucleotide, alicaforsen (ISIS 2302), in active steroid dependent Crohn's disease. Gut *51*, 30–36.

121. Yu, R.Z., Wang, Y.F., Norris, D.A., Kim, T.W., Narayanan, P., Geary, R.S., Monia, B.P., and Henry, S.P. (2020). Immunogenicity Assessment of Inotersen, a 2 '-O-(2-Methoxyethyl) Antisense Oligonucleotide in Animals and Humans: Effect on Pharmacokinetics, Pharmacodynamics, and Safety. Nucleic Acid Therapeut. *30*, 265–275.

122. (2022). MedDRA® the Medical Dictionary for Regulatory Activities terminology is the international medical terminology developed under the auspices of the International Council for Harmonisation of Technical Requirements for Pharmaceuticals for Human Use (ICH) (MedDRA® Trademark Is Registered by ICH).

123. Imrey, P.B. (2020). Limitations of Meta-analyses of Studies With High Heterogeneity. JAMA Netw. Open *3*, e1919325.

124. Haldane, J.B. (1956). The estimation and significance of the logarithm of a ratio of frequencies. Ann. Hum. Genet. *20*, 309–311.

125. Sewing, S., Roth, A.B., Winter, M., Dieckmann, A., Bertinetti-Lapatki, C., Tessier, Y., McGinnis, C., Huber, S., Koller, E., Ploix, C., et al. (2017). Assessing single-stranded oligonucleotide drug-induced effects in vitro reveals key risk factors for thrombocytopenia. PLoS One *12*, e0187574.

126. Flierl, U., Nero, T.L., Lim, B., Arthur, J.F., Yao, Y., Jung, S.M., Gitz, E., Pollitt, A.Y., Zaldivia, M.T.K., Jandrot-Perrus, M., et al. (2015). Phosphorothioate backbone modifications of nucleotide-based drugs are potent platelet activators. J. Exp. Med. *212*, 129–137.

127. Zaslavsky, A., Adams, M., Cao, X., Yamaguchi, A., Henderson, J., Busch-Østergren, P., Udager, A., Pitchiaya, S., Tourdot, B., Kasputis, T., et al. (2021). Antisense oligonucleotides and nucleic acids generate hypersensitive platelets. Thromb. Res. *200*, 64–71.

128. van Putten, M., Young, C., van den Berg, S., Pronk, A., Hulsker, M., Karnaoukh, T. G., Vermue, R., van Dijk, K.W., de Kimpe, S., and Aartsma-Rus, A. (2014). Preclinical studies on intestinal administration of antisense oligonucleotides as a model for oral delivery for treatment of duchenne muscular dystrophy. Mol. Ther. Nucleic Acids *3*, e211.

129. Iversen, P.L. (2016). Structure activity study of clinically observed adverse events and oligomer chemistry. J. Drug Discov. Dev. Deliv. *3*.

130. Crooke, S.T., Witztum, J.L., Bennett, C.F., and Baker, B.F. (2018). RNA-Targeted Therapeutics. Cell Metab. *27*, 714–739.

131. Wu, B., Lu, P., Benrashid, E., Malik, S., Ashar, J., Doran, T.J., and Lu, Q.L. (2009). Dose-dependent restoration of dystrophin expression in cardiac muscle of dystrophic mice by systemically delivered morpholino. Gene Ther. *17*, 132–140.

132. Heald, A.E., Iversen, P.L., Saoud, J.B., Sazani, P., Charleston, J.S., Axtelle, T., Wong, M., Smith, W.B., Vutikullird, A., and Kaye, E. (2014). Safety and pharmacokinetic profiles of phosphorodiamidate morpholino oligomers with activity against ebola virus and marburg virus: results of two single-ascending-dose studies. Antimicrob. Agents Chemother. *58*, 6639–6647.

133. Levin, A.A. (1999). A review of issues in the pharmacokinetics and toxicology of phosphorothioate antisense oligonucleotides. Biochim. Biophys. Acta *1489*, 69–84.

134. (2023). A Randomised, Single-Blind, Single Center, Placebo-Controlled, Phase I Study to Assess the Safety, Tolerability, and Pharmacokinetics of AZD7503 Following Multiple Subcutaneous Dose Administration in Healthy Japanese Participants (-AstraZeneca). https://www.astrazenecaclinicaltrials.com/study/D9230C00005/.

135. Brannagan, T.H., Coelho, T., Wang, A.K., Polydefkis, M.J., Dyck, P.J., Berk, J.L., Drachman, B., Gorevic, P., Whelan, C., Conceição, I., et al. (2022). Long-term efficacy and safety of inotersen for hereditary transthyretin amyloidosis: NEURO-TTR open-label extension 3-year update. J. Neurol. *269*, 6416–6427.

136. Cheung, T.T., Luk, A.O.Y., Zhang, Y., Pavlovic, V., Wat, C., Das, S., Surujbally, B., Triyatni, M., and Grippo, J.F. (2023). A single ascending dose study of single-stranded oligodeoxyribonucleotide RO7062931 in Chinese healthy volunteers. Clin. Transl. Sci. *16*, 1272–1282.

137. Clemens, P.R., Rao, V.K., Connolly, A.M., Harper, A.D., Mah, J.K., McDonald, C. M., Smith, E.C., Zaidman, C.M., Nakagawa, T., and Hoffman, E.P. (2023). Efficacy and Safety of Viltolarsen in Boys With Duchenne Muscular Dystrophy: Results From the Phase 2, Open-Label, 4-Year Extension Study. J. Neuromuscul. Dis. *10*, 439–447.

138. Finkel, R.S., Day, J.W., Pascual Pascual, S.I., Ryan, M.M., Mercuri, E., De Vivo, D. C., Montes, J., Gurgel-Giannetti, J., Monine, M., Gambino, G., et al. (2023). DEVOTE Study Exploring Higher Dose of Nusinersen in Spinal Muscular Atrophy: Study Design and Part A Results. J. Neuromuscul. Dis. *10*, 813–823.

139. Gale, D.P., Gross, O., Wang, F., Esteban de la Rosa, R.J., Hall, M., Sayer, J.A., Appel, G., Hariri, A., Liu, S., Maski, M., et al.; HERA Clinical Trial Group (2024). A Randomized Controlled Clinical Trial Testing Effects of Lademirsen on Kidney Function Decline in Adults with Alport Syndrome. Clin. J. Am. Soc. Nephrol. *19*, 995–1004.

140. Han, K., Theodore, D., McMullen, G., Swayze, E., McCaleb, M., Billioud, G., Wieland, S., Hood, S., Paff, M., Bennett, C.F., et al. (2022). Preclinical and Phase 1 Assessment of Antisense Oligonucleotide Bepirovirsen in Hepatitis B Virus-Transgenic Mice and Healthy Human Volunteers: Support for Clinical Dose Selection and Evaluation of Safety, Tolerability, and Pharmacokinetics of Single and Multiple Doses. Clin. Pharmacol. Drug Dev. *11*, 1191–1202.

141. Karwatowska-Prokopczuk, E., Lesogor, A., Yan, J.H., Hoenlinger, A., Margolskee, A., Li, L., and Tsimikas, S. (2024). Efficacy and safety of olezarsen in lowering apolipoprotein C-III and triglycerides in healthy Japanese Americans. Lipids Health Dis. *23*, 329.

142. Karwatowska-Prokopczuk, E., Lesogor, A., Yan, J.H., Hurh, E., Hoenlinger, A., Margolskee, A., Xia, S., and Tsimikas, S. (2023). Efficacy and safety of pelacarsen in lowering Lp(a) in healthy Japanese subjects. J. Clin. Lipidol. *17*, 181–188.

143. Komaki, H., Takeshita, E., Kunitake, K., Ishizuka, T., Shimizu-Motohashi, Y., Ishiyama, A., Sasaki, M., Yonee, C., Maruyama, S., Hida, E., et al. (2025). Phase 1/2 trial of brogidirsen: Dual-targeting antisense oligonucleotides for exon 44 skipping in Duchenne muscular dystrophy. Cell Rep. Med. *6*, 101901.

144. Lightbourne, M., Startzell, M., Bruce, K.D., Brite, B., Muniyappa, R., Skarulis, M., Shamburek, R., Gharib, A.M., Ouwerkerk, R., Walter, M., et al. (2022). Volanesorsen, an antisense oligonucleotide to apolipoprotein C-III, increases lipoprotein lipase activity and lowers triglycerides in partial lipodystrophy. J. Clin. Lipidol. *16*, 850–862.

145. McCaleb, M.L., Hughes, S.G., Grossman, T.R., Frazer-Abel, A., Jung, B., Yin, L., Henry, S.P., Monia, B.P., Schneider, E., Geary, R., et al. (2025). Inhibiting the alternative pathway of complement by reducing systemic complement factor B: Randomized, double-blind, placebo-controlled phase 1 studies with Sefaxersen. Immunobiology *230*, 152876.

146. Mercuri, E., Seferian, A.M., Servais, L., Deconinck, N., Stevenson, H., Ni, X., Zhang, W., East, L., Yonren, S., Muntoni, F., et al. (2023). Safety, tolerability and pharmacokinetics of eteplirsen in young boys aged 6-48 months with Duchenne muscular dystrophy amenable to exon 51 skipping. Neuromuscul. Disord. *33*, 476–483.

147. Miller, T.M., Cudkowicz, M.E., Genge, A., Shaw, P.J., Sobue, G., Bucelli, R.C., Chiò, A., Van Damme, P., Ludolph, A.C., Glass, J.D., et al. (2022). Trial of Antisense Oligonucleotide Tofersen for SOD1 ALS. N. Engl. J. Med. *387*, 1099–1110.

148. Nishina, T., Fujita, T., Yoshizuka, N., Sugibayashi, K., Murayama, K., and Kuboki, Y. (2022). Safety, tolerability, pharmacokinetics and preliminary antitumour activity of an antisense oligonucleotide targeting STAT3 (danvatirsen) as monotherapy and in combination with durvalumab in Japanese patients with advanced solid malignancies: a phase 1 study. BMJ Open *12*, e055718.

149. Oral, E.A., Garg, A., Tami, J., Huang, E.A., O'Dea, L.S.L., Schmidt, H., Tiulpakov, A., Mertens, A., Alexander, V.J., Watts, L., et al. (2022). Assessment of efficacy and safety of volanesorsen for treatment of metabolic complications in patients with familial partial lipodystrophy: Results of the BROADEN study: Volanesorsen in FPLD; The BROADEN Study. J. Clin. Lipidol. *16*, 833–849.

150. Riedl, M.A., Tachdjian, R., Lumry, W.R., Craig, T., Karakaya, G., Gelincik, A., Stobiecki, M., Jacobs, J.S., Gokmen, N.M., Reshef, A., et al. (2024). Efficacy and Safety of Donidalorsen for Hereditary Angioedema. N. Engl. J. Med. *391*, 21–31.

151. Stroes, E.S.G., Alexander, V.J., Karwatowska-Prokopczuk, E., Hegele, R.A., Arca, M., Ballantyne, C.M., Soran, H., Prohaska, T.A., Xia, S., Ginsberg, H.N., et al. (2024). Olezarsen, Acute Pancreatitis, and Familial Chylomicronemia Syndrome. N. Engl. J. Med. *390*, 1781–1792.

152. Tassone, P., Di Martino, M.T., Arbitrio, M., Fiorillo, L., Staropoli, N., Ciliberto, D., Cordua, A., Scionti, F., Bertucci, B., Salvino, A., et al. (2023). Safety and activity of the first-in-class locked nucleic acid (LNA) miR-221 selective inhibitor in refractory advanced cancer patients: a first-in-human, phase 1, open-label, dose-escalation study. J. Hematol. Oncol. *16*, 68.

153. Thornton, C.A., Moxley, R.T., 3rd, Eichinger, K., Heatwole, C., Mignon, L., Arnold, W.D., Ashizawa, T., Day, J.W., Dent, G., Tanner, M.K., et al. (2023). Antisense oligonucleotide targeting DMPK in patients with myotonic dystrophy type 1: a multicentre, randomised, dose-escalation, placebo-controlled, phase 1/2a trial. Lancet Neurol. *22*, 218–228.

154. Winkelmayer, W.C., Lensing, A.W.A., Thadhani, R.I., Mahaffey, K.W., Walsh, M., Pap, Á.F., Willmann, S., Thelen, K., Hodge, S., Solms, A., et al. (2024). A Phase II randomized controlled trial evaluated antithrombotic treatment with fesomersen in patients with kidney failure on hemodialysis. Kidney Int. *106*, 145–153.

155. Witztum, J.L., Gaudet, D., Arca, M., Jones, A., Soran, H., Gouni-Berthold, I., Stroes, E.S.G., Alexander, V.J., Jones, R., Watts, L., et al. (2023). Volanesorsen and triglyceride levels in familial chylomicronemia syndrome: Long-term efficacy and safety data from patients in an open-label extension trial. J. Clin. Lipidol. *17*, 342–355.

156. Woodcock, I.R., Tachas, G., Desem, N., Houweling, P.J., Kean, M., Emmanuel, J., Kennedy, R., Carroll, K., de Valle, K., Adams, J., et al. (2024). A phase 2 open-label study of the safety and efficacy of weekly dosing of ATL1102 in patients with non-ambulatory Duchenne muscular dystrophy and pharmacology in mdx mice. PLoS One *19*, e0294847.

157. Yuen, M.F., Heo, J., Kumada, H., Suzuki, F., Suzuki, Y., Xie, Q., Jia, J., Karino, Y., Hou, J., Chayama, K., et al. (2022). Phase IIa, randomised, double-blind study of GSK3389404 in patients with chronic hepatitis B on stable nucleos(t)ide therapy. J. Hepatol. *77*, 967–977.

158. Yuen, M.F., Lim, S.G., Plesniak, R., Tsuji, K., Janssen, H.L.A., Pojoga, C., Gadano, A., Popescu, C.P., Stepanova, T., Asselah, T., et al. (2022). Efficacy and Safety of Bepirovirsen in Chronic Hepatitis B Infection. N. Engl. J. Med. *387*, 1957–1968.

159. Nikles, J., Onghena, P., Vlaeyen, J.W.S., Wicksell, R.K., Simons, L.E., McGree, J.M., and McDonald, S. (2021). Establishment of an International Collaborative Network for N-of-1 Trials and Single-Case Designs. Contemp. Clin. Trials Commun. *23*, 100826.

160. Page, M.J., Moher, D., Bossuyt, P.M., Boutron, I., Hoffmann, T.C., Mulrow, C.D., Shamseer, L., Tetzlaff, J.M., Akl, E.A., Brennan, S.E., et al. (2021). PRISMA 2020 explanation and elaboration: updated guidance and exemplars for reporting systematic reviews. BMJ *372*, n160.

161. Ouzzani, M., Hammady, H., Fedorowicz, Z., and Elmagarmid, A. (2016). Rayyan-a web and mobile app for systematic reviews. Syst. Rev. *5*, 210.

162. Oberemok, V.V., Laikova, K.V., Repetskaya, A.I., Kenyo, I.M., Gorlov, M.V., Kasich, I.N., Krasnodubets, A.M., Gal'chinsky, N.V., Fomochkina, I.I., Zaitsev, A.S., et al. (2018). A Half-Century History of Applications of Antisense Oligonucleotides in Medicine, Agriculture and Forestry: We Should Continue the Journey. Molecules *23*, 1302.

163. Aartsma-Rus, A., and Takeda, S. (2025). A historical perspective on the development of antisense oligonucleotide treatments for Duchenne muscular dystrophy and spinal muscular atrophy. J. Neuromuscul. Dis. 22143602251317422.

164. Thakur, S., Sinhari, A., Jain, P., and Jadhav, H.R. (2022). A perspective on oligonucleotide therapy: Approaches to patient customization. Front. Pharmacol. *13*, 1006304.

165. Weber, F., Knapp, G., Ickstadt, K., Kundt, G., and Glass, Ä. (2020). Zero-cell corrections in random-effects meta-analyses. Res. Synth. Methods *11*, 913–919.

OMTN, Volume *37*

Supplemental information

Meta-analysis of adverse events in clinical studies with antisense oligonucleotide therapies

Cisse Vermeer, Rindert R. Venema, Erwin Birnie, Marieke C. Bolling, Nine Knoers, Jeroen Bremer, and Peter C. van den Akker

PICO+S based search strategy as used for Pubmed. The inclusion of specific drugs in the search strategy did not guarantee the inclusion of relevant articles into the dataset.

("Oligonucleotides"[Mesh] OR "Oligonucleotide Probes"[Mesh] OR “oligonucleotide*”[tiab] OR “oligodeoxyribonucleotide*”[tiab] OR “oligoribonucleotide*”[tiab] OR “nusinersen”[tiab] OR “fomivirsen”[tiab] OR “pegaptanib”[tiab] OR “defibrotide”[tiab] OR “inotersen”[tiab] OR “eteplirsen”[tiab] OR “mipomersen”[tiab])
AND
("Drug-Related Side Effects and Adverse Reactions"[Mesh] OR "Long Term Adverse Effects"[Mesh] OR "adverse effects" [Subheading] OR “adverse*”[tiab] OR “safety”[tiab] OR “efficac*”[tiab] OR (“benefit”[tiab] *AND* “risk”[tiab]) OR "complications"[Subheading] OR "complication*"[tiab] OR "complicating"[tiab] OR "side effect*”[tiab] OR "contraindication*"[tiab] OR "contraindicated"[tiab] OR “toxicity”[tiab] OR "drug eruption*"[tiab] OR "toxic effect*"[tiab] OR "Toxic Actions"[Mesh] OR “toxic action*”[tiab] OR "poisoning"[tiab] OR "chemically induced"[Subheading] OR "chemically induced"[tiab] OR "Dose-Response Relationship, Drug"[Mesh] OR "Dose-Response Relationship“[tiab] OR “Treatment Failure”[tiab] OR "Treatment Failure"[Mesh])
AND
("Clinical Trial" [Publication Type] OR trial*[ti] OR “clinical study”[tiab] OR “clinical trial”[tiab])
NOT
("animals"[MeSH] NOT "humans"[MeSH])

Table S1, Significant differences in incidence rates between RNAse H and splice alteration adverse events with the respective 95% confidence intervals (95% CI). NEC: Not elsewhere classifiable.

ADVERSE EVENT	SPLICE STUDIES	SPLICE PREVALENCE	95% CI LOWER	95% CI UPPER	RNASE H STUDIES	RNASE H PREVALENCE	95% CI LOWER	95% CI UPPER
ASTHENIC CONDITIONS	3	0.10	0.04	0.23	38	0.30	0.23	0.38
HEADACHES NEC	15	0.27	0.22	0.33	31	0.17	0.14	0.21
UPPER RESPIRATORY TRACT INFECTIONS	17	0.32	0.21	0.45	21	0.01	0.00	0.06
COUGHING AND ASSOCIATED SYMPTOMS	14	0.29	0.20	0.40	11	0.09	0.07	0.13
COAGULATION AND BLEEDING ANALYSES	4	0.05	0.02	0.14	7	0.53	0.31	0.73
UPPER RESPIRATORY TRACT SIGNS AND SYMPTOMS	9	0.20	0.12	0.31	5	0.07	0.04	0.12
POTASSIUM IMBALANCE	3	0.41	0.25	0.59	3	0.12	0.07	0.20
RATE AND RHYTHM DISORDERS NEC	8	0.10	0.06	0.18	3	0.02	0.01	0.06
VIRAL INFECTIONS NEC	7	0.16	0.09	0.27	3	0.05	0.04	0.07
DERMATITIS AND ECZEMA	13	0.14	0.10	0.21	2	0.02	0.01	0.09

Table S2, Incidence of all reported events in three or more studies resulting from ASO treatment. with the respective 95% confidence intervals (95% CI). NEC: Not elsewhere classifiable

For Table S2 see supplemental spreadsheet *TableS2.xlsx*

Table S3, ***Risk of bias analysis per article***. *Bias assessment per category of bias is made with an assessment of low (green) some (orange) of high (red) risk of bias. Entries marked with * were separately assessed because of different study formats conducted within the same article*

For Table S3 see supplemental spreadsheet *TableS3.xlsx*

***Table S4**, **Variables used in the meta regression that were performed for each analysis.** In each analysis a study was assigned a group for every variable.*

REGRESSION VARIABLE	GROUP 1	GROUP 2	GROUP 3	GROUP 4	GROUP 5
**CHEMISTRY**	2'-Ome-PS	2'-MOE-PS	DNA oligonucleotides	PMO	Other chemistries
**SUBJECT HEALTH**	Healthy	Cancer	Muscle diseases	Eye diseases	Other disease
**ADMINISTRATION ROUTE**	Intravenous	Intrathecal	Subcutaneous	Other administration routes	
**MODE OF ACTION**	RNAse H	Splice switching	Other mode of action		
**SUBJECT AGE**	0-18	19-64	65+		
**PUBLICATION DATE**	1990-2010	2011-2017	2018-2023		
**PLACEBO CONTROLLED**	Yes	No			

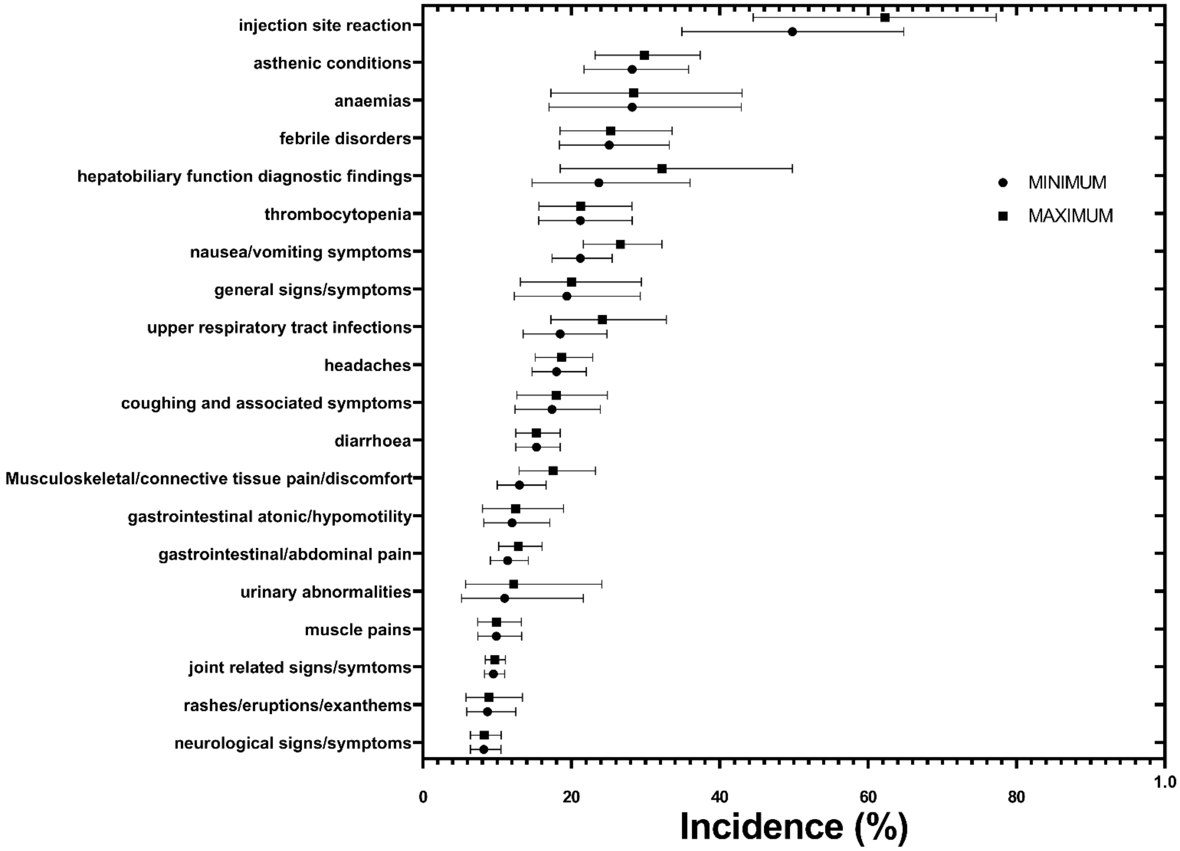

Figure S1, Differences in incidence rates of the top 20 most often reported adverse events*, comparing minimum and maximum event counts. There are no significant differences between minimum and maximum counts for any of the measured events.*

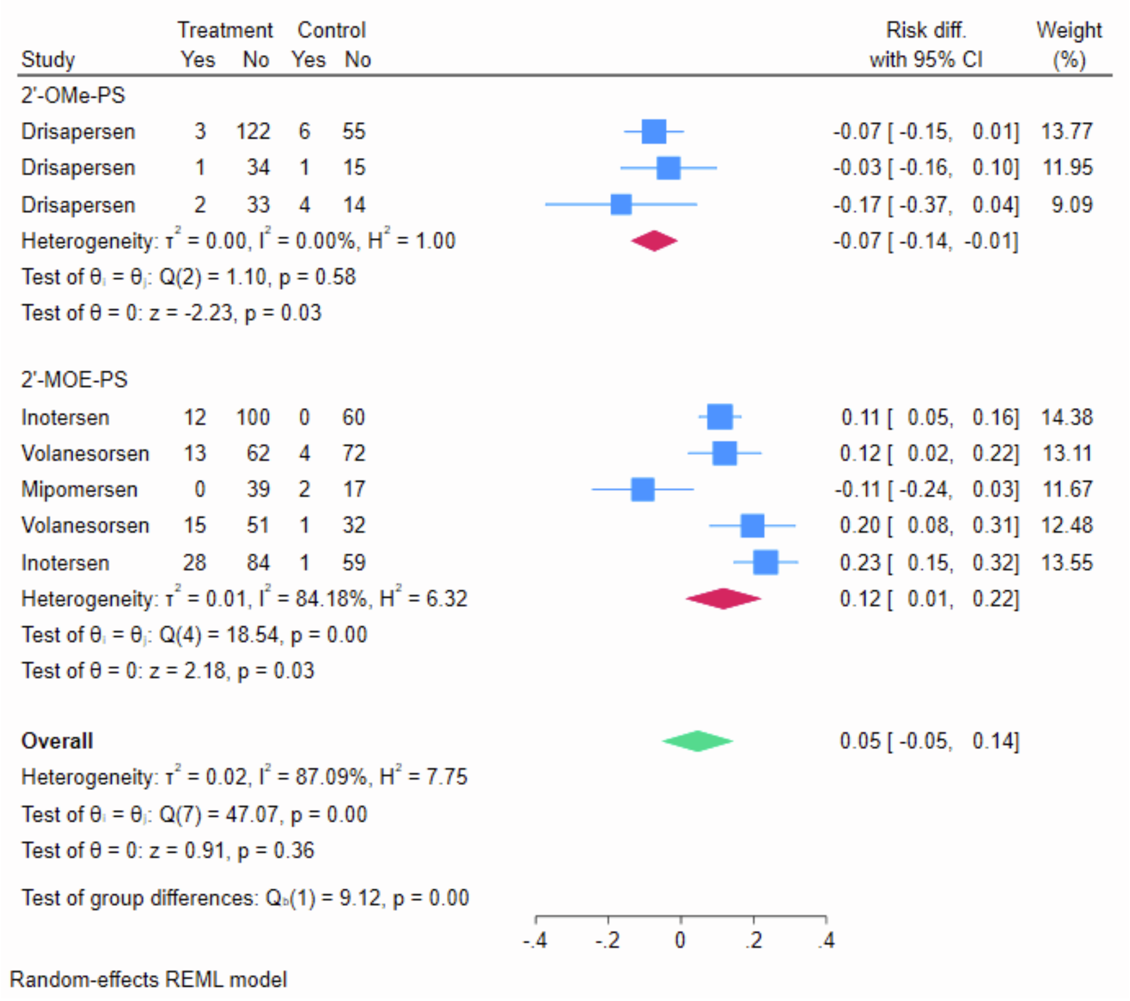

Figure S2, Forest plot of merged thrombocytopenia-related AEs. *These AEs include the terms "platelet disorders", "platelet disorders NEC (Not elsewhere clarified)", "thrombocytopenia", "platelet analyses" and "coagulation and bleeding analyses". Overall effect size is shown by the green diamond. Effect sizes per ASO chemistry subgroup are shown as red diamonds, demonstrating that coagulation-related events have a statistically significant higher risk difference in patient groups treated with 2'MOE PS ASOs compared to 2'OMe PS ASOs. The effect size per study is presented as a blue box with corresponding confidence intervals (95% CI). This forest plot shows that the overall effect size is not significant (θi: $p>0.05$) but the effect sizes for each subgroup are significant (θ: $p<0.05$). Measures of heterogeneity (T^2, I^2, H^2) point to show reduced heterogeneity in each of the subgroups.*
